# Molecular dynamics study of tropical calcific pancreatitis (TCP) associated calcium-sensing receptor single nucleotide variation

**DOI:** 10.3389/fmolb.2022.982831

**Published:** 2022-10-04

**Authors:** Ashish Shrivastava, Kartavya Mathur, Rohit Kumar Verma, Sri Krishna Jayadev Magani, Deepak Krishna Vyas, Ashutosh Singh

**Affiliations:** ^1^ Translational Bioinformatics and Computational Genomics Research Lab, Department of Life Sciences, Shiv Nadar Institution of Eminence, Gautam Buddha Nagar, UP, India; ^2^ Cancer Biology Lab, Department of Life Sciences, Shiv Nadar Institution of Eminence, Gautam Buddha Nagar, UP, India; ^3^ Department of Biotechnology, Lachoo Memorial College of Science and Technology, Jodhpur, RJ, India

**Keywords:** CaSR, calcium sensing receptor, single nucleotide variants, tropical calcific pancreatitis, pancreatitis, mutTCPdb, molecular dynamics simulation

## Abstract

Tropical Calcific Pancreatitis (TCP) is a chronic non-alcoholic pancreatitis characterised by extensive calcification. The disease usually appears at a younger age and is more common in tropical regions. This disease’s progression can lead to pancreatic diabetes, which can subsequently lead to pancreatic cancer. The CASR gene encodes a calcium-sensing receptor (CaSR), which is a GPCR protein of class C. It is expressed in the islets of Langerhans, the parathyroid gland, and other tissues. It primarily detects small gradients in circulating calcium concentrations and couples this information to intracellular signalling, which helps to regulate PTH (parathyroid hormone) secretion and mineral ion homeostasis. From co-leading insulin release, CaSR modulates ductal HCO_3−_ secretion, Ca^2+^ concentration, cell-cell communication, β-cell proliferation, and intracellular Ca^2+^ release. In pancreatic cancer, the CaSR limits cell proliferation. TCP-related four novel missense mutations P163R, I427S, D433H and V477A, found in CaSR extracellular domain (ECD) protein, which were reported in the mutTCPdb Database (https://lms.snu.edu.in/mutTCPDB/index.php). P163R mutation occurs in ligand-binding domain 1 (LBD-1) of the CaSR ECD. To investigate the influence of these variations on protein function and structural activity multiple in-silico prediction techniques such as SIFT, PolyPhen, CADD scores, and other methods have been utilized. A 500 ns molecular dynamic simulation was performed on the CaSR ECD crystal structure and the corresponding mutated models. Furthermore, Principal Component Analysis (PCA) and Essential Dynamics analysis were used to forecast collective motions, thermodynamic stabilities, and the critical subspace crucial to CaSR functions. The results of molecular dynamic simulations showed that the mutations P163R, I427S, D433H, and V477A caused conformational changes and decreased the stability of protein structures. This study also demonstrates the significance of TCP associated mutations. As a result of our findings, we hypothesised that the investigated mutations may have an effect on the protein’s structure and ability to interact with other molecules, which may be related to the protein’s functional impairment.

## 1 Introduction

Inflammation of the pancreas is referred to as pancreatitis. Pancreatitis can be classified as acute or chronic (Whitcomb, 1999). Tropical Calcific Pancreatitis (TCP) is a juvenile form of chronic calcific pancreatitis. It is only found in countries of the tropical world such as India, Bangladesh, Sri Lanka, Nigeria, Uganda, Africa, Brazil, and Thailand (Barman et al., 2003; Choudhuri et al., 2008; Cyriac et al., 2012; Kangas-Dick et al., 2016; Tan et al., 1980; Witt and Bhatia, 2008). Abdominal pressure, ductal dilatation, large pancreatic calculi, and pancreatic atrophy are all hallmarks of TCP (Lee and Raleigh, 2011; Paliwal et al., 2014). Progression of TCP may culminate in the development of fibro-calculus pancreatitis (FCPD), a kind of diabetes that occurs as the disease advances to its severe stages (Kibirige et al., 2012; Mohan, 1998; Unnikrishnan and Mohan, 2015; Yajnik and Shelgikar, 1993). TCP is diagnosed by the development of large pancreatic calculi, which can be detected via endoscopic retrograde cholangiopancreatography-computed tomography (Kangas-Dick et al., 2016; Barman et al., 2003; Chari et al., 1994). Continued progression of this illness increases the likelihood of pancreatic cancer and, as a result, death from late discovery. Pancreatic cancer and TCP both have an unclear aetiology (Chari et al., 1994; Barman et al., 2003). Pancreatic cancer is the seventh leading cause of mortality globally, the 10th leading cause of death in India, and the 5th leading cause of death in the United States, according to reports (Fitzmaurice et al., 2017). Hence early detection of TCP can reduce the burden of pancreatic cancer.

Several gene SNVs associated with TCP have been reported in the mutTCPdb database. (Singh et al., 2018). Among these genes, one of the most prominent, CASR gene, was discovered to be involved in TCP with four non-synonymous coding SNVs (Murugaian et al., 2008; Paliwal et al., 2014). These SNVs are located in exons 3, 4, and 5 of the CASR gene and result in P163R, I427S, D433H, and V477A protein sequence alterations. The CASR gene encodes a calcium-sensing receptor (CaSR), a GPCR protein belonging to the class C subfamily. GPCR Class C family comprises Calcium-sensing receptor, Metabotropic glutamate receptor, GABA_B_, and Taste 1 receptors (Supplementary Figure S1; source: GPCRdb). A phylogenetic analysis of class C GPCRs with G-protein coupling and associated ligand types is shown in Supplementary Figure S2 (source: GPCRdb) (Isberg et al., 2016; Kooistra et al., 2021).

Calcium molecules bind to CaSR, allowing it to monitor and regulate the quantity of calcium in the bloodstream. The receptor is activated when a specific concentration of calcium is reached, and the active receptor sends out signals to cease actions that raise the amount of calcium in the blood. It detects minor changes in circulating calcium concentration and couples these details to intracellular signalling, resulting in the regulation of parathyroid hormone (PTH) secretion and mineral ion homeostasis (Xie et al., 2014).

The CASR gene is highly expressed in the parathyroid gland and renal tubules. Calcium reabsorption from filtered fluids is blocked by increased calcium binding to CaSR in renal cells. At the same time, expression is shown in the pancreas, islets of Langerhans, gut, skin, brain, heart, bone, lung, and other tissues (Rácz et al., 2002; Pidasheva et al., 2004; Hendy and Canaff, 2016). CaSR plays a variety of physiological roles in digestive processes such as gastric secretion, insulin release, secretion/absorption, and fluid transport, as well as inducing cell apoptosis in hepatic injury. CaSR activation can affect ductal HCO_3-_ secretion and Ca^2+^ concentration in pancreatic juice. CaSR could stimulate cell-cell communication, β-cell proliferation, and intracellular Ca^2+^ release to co-lead insulin secretion. CaSR also inhibits cell proliferation in pancreatic cancer, and CaSR mutations, with or without SPINK1, cause pancreatitis (Pidasheva et al., 2004).

**FIGURE 1:**
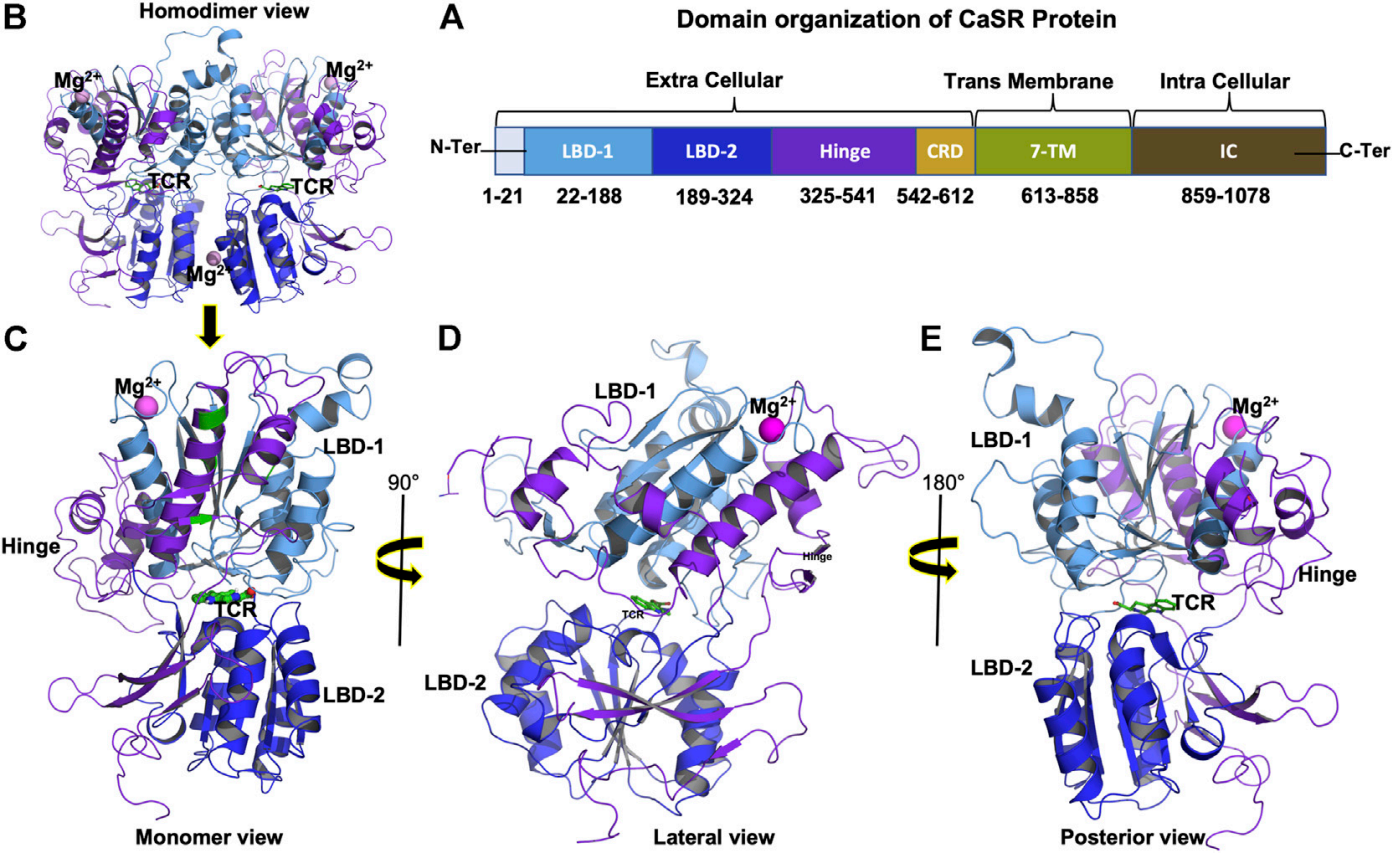
**(A)** Domain organization of CaSR protein showing extracellular, transmembrane, and intracellular domain boundaries, **(B)** 3D structure of CaSR Extra Cellular Domain (ECD) homodimer (PDB ID: 5FBK), **(C)** Monomer 3D structure of CaSR ECD, **(D)** Monomer 3D structure of CaSR ECD lateral view (90°), and **(E)** Monomer 3D structure of CaSR ECD posterior view (180°). Figure **(B)** shows CaSR protein structure bound with metal ions Mg2+ (magenta) in LBD1 and at the interface of homodimernear LBD2. Figure **(B)** also shows Co-agonist L-tryptophan (TCR) (green) bound at the interface of LBD1 and LBD2. Domains in figure **(B)**, **(C)**, **(D)**, and **(E)** are represented as per color scheme used in figure 1 **(A)**.

In a previous study, the CASR gene was identified as a potential candidate gene in TCP, and combining a SPINK1 gene mutation with a CASR gene mutation can enhance the risk of TCP. CASR or SPINK1 genes SNV could raise the risk of developing chronic pancreatitis (CP). Since high intracellular calcium levels activate trypsinogen within acinar cells, which results in pancreatic autodigestion and pancreatitis (Murugaian et al., 2008; Xie et al., 2014). CaSR mutations are most commonly associated with hereditary hypocalciuric hypercalcemia, extreme hyperparathyroidism in neonates, and autosomal dominant hypocalcemia (Pidasheva et al., 2004; Vahe et al., 2017). CaSR variation can result in faulty receptors that are unable to detect calcium levels, resulting in the production of massive calculi, which is a common symptom of Tropical Calcific Pancreatitis. CaSR is a 1,078 amino acid long protein with an N-terminal signal peptide, an extracellular domain (ECD), a seven-transmembrane (7TM) domain, and an intracellular domain (ICD) (Figure 1) (Muto et al., 2007; Hendy and Canaff, 2016). The extracellular domain (ECD) is further subdivided into two ligand-binding domains (LBD1 and LBD2) and one cysteine rich (CR) domain (Geng et al., 2016). The Venus flytrap domain, which includes the orthosteric binding site for its ligand, is formed by LBD1 and LBD2. The CaSR forms a homodimer, and both protomers are in open conformation at rest. Anion (such as PO_4_^3-^) binding promotes the inactive conformation with an open cleft between both protomers. In the active state, both protomers approach each other and form a closed conformation in the association of agonist binding (Zhang et al., 2016b; Geng et al., 2016). CaSR contains four Ca^2+^ binding sites, one of which is located between the homodimer interface and the other three of which are located in ECD (Silve et al., 2005).

When the agonist binds to the CaSR, it causes ECD to undergo large conformational changes and begin moving toward each protomer, resulting in a closed-closed conformation. These modifications then result in signalling toward the 7TM domain. Because amino acid ligand alone is insufficient to activate the receptor, metal ions Ca^2+^ or Mg^2+^ are required for full activation, but the presence of metal ions alone can promote receptor activation (Zhang et al., 2016a; Geng et al., 2016; Ling et al., 2021).

The association of genotypic variation at the nucleotide level could alter the 3D structure and functions of proteins, making genomics increasingly important for human health. Single nucleotide variations (SNVs) play a significant role in phenotypic changes when they occur in the coding DNA sequence of a biologically important gene, resulting in a non-synonymous missense effect and a single amino acid change in the protein sequence of the respective gene (Sunyaev et al., 2000; Bhattacharya et al., 2017).

A molecular dynamics simulation study of protein can provide insight into the details of atomic behaviour of protein upon mutation in protein structure (Singh et al., 2019; Chitongo et al., 2020; Shuaib et al., 2020; Navapour and Mogharrab, 2021; Yadav and Singh, 2021). As a result, motion analysis can be used to investigate the stability and changes in the dynamic behaviour of proteins because of mutation. The essential dynamics (ED) analysis, also known as PCA (principal component analysis), is useful for analysing the most concerted motions available from MD simulation trajectories (Amadei et al., 1993; Amir et al., 2019). A comparison of the essential dynamics of WT and mutant proteins can provide an additional measure for studying the impact of the mutation on protein stability and functional changes.

This study investigated the effect of CASR gene SNVs associated with TCP on protein structure and function. These SNVs leads to mutations in protein sequence such as P163R, I427S, D433H, and V477A (Murugaian et al., 2008).

SIFT, PolyPhen, CADD, PROVEAN, I-Mutant, and PANTHER, along with other methods, were used to predict the impact of SNV on structure and function. Using these methods, all four mutations predicted to have a significant impact on protein structure and function. To investigate the atomic-level details and subsequent impact of SNVs on structure and functions, 500 ns molecular dynamics simulations of WT and mutant proteins were performed. In addition, essential dynamics analysis for MD simulation trajectories was performed to investigate the most concerted motion of the protein as well as the effect of the mutation.

Based on the analysis we can summarize, out of P163R, I427S, D433H, and V477A mutations P163R is destabilizing and having significant impact on structure and function. This mutation significantly impact protein structure evaluated using various in silico measures, MD simulation, and essential dynamics analysis. However, MD simulation studies of all mutations also showed deviations from WT stability and dynamics.

## 2 Materials and methods

### 2.1 Data collection

The CASR SNV data associated with TCP were retrieved from the mutTCPdb database (https://lms.snu.edu.in/mutTCPDB/index.php) (Singh et al., 2018). This database consists 100 of TCP associated SNVs found in 11 genes from the literature search. SNVs with TCP IDs tcp8461, tcp8462, tcp8463, and tcp8464 were used in this analysis and subsequent mutations in protein sequence were P163R, I427S, D433H, and V477A respectively (Murugaian et al., 2008; Paliwal et al., 2014).

#### 2.1 1 In-silico non-synonymous SNP analysis

The non-synonymous SNP analysis was carried out to predict mutation’s functional impact using various in-silico methods and servers. These are categorized into sequence based, structure based, and pathogenecity based methods.

#### 2.1 2 Sequence-based methods

CADD (combined annotation dependent depletion) is a web-based server that predicts the deleteriousness of variants across the human genome (Rentzsch et al., 2019). Using this web server’s single nucleotide variant (SNV) lookup form, scoring and annotation of single nucleotide variant at the specific location were accessed for SIFT (Sorting Intolerant from Tolerant), PolyPhen (Polymorphism Phenotyping) and PHRED (https://cadd.gs.washington.edu/snv).

SIFT is an algorithm that predicts whether amino acid substitution will affect protein function based on amino acid sequence homologies and physical properties. A SIFT score < 0.05 is predicted to be harmful. A substitution with a score ≥ 0.05 is predicted to be tolerated. PHRED score is a scaled C-score of ≥10, indicating that these are predicted to be the 10% most deleterious substitutions that can be made to the human genome. A score of ≥20 denotes the 1% most deleterious mutation, and so on. PolyPhen is a tool that predicts the possible impact of amino acid substitution on the structure and function of a human protein based on physical and comparative considerations. It predicts whether the substitution is “probably damaging”, “possibly damaging”, or “benign”.

PROVEAN (Protein Variation Effect Analyzer) is a web-based tool that predicts whether an amino acid substitution affects the biological function of the CaSR protein (http://provean.jcvi.org/). PROVEAN’s capacity to predict single amino acid alterations was compared to that of the existing tool CADD in this study (SHIFT, PolyPhen-2, and Phred quality score). A PROVEAN value of 2.5 or higher is regarded neutral, whereas a score of less than 2.5 is considered detrimental for a nsSNP (Choi and Chan, 2015).

The PON-P2 (http://structure.bmc.lu.se/PON-P2/) tool predicts the pathogenicity of amino acid substitutions; it is a machine learning-based classifier for the classification of amino acid features on human proteins. PON-P2 divides amino acid variants into three categories: pathogenic, neutral, and unknown. It also makes use of Gene Ontology (GO) annotations, evolutionary conservation, and, if available, annotations of functional sites. This tool can analyse various formats of nsSNP and is easily accessible via amino acid substitution(s) and one of Ensembl gene identifiers, UniProtKB/accession ID, for identifier submission (Niroula et al., 2015).

PANTHER (Protein Analysis Through Evolutionary Relationships) (http://www.pantherdb.org/about.jsp) is a protein classification system designed to facilitate high-throughput analysis by classifying proteins (and their genes). It predicts the likelihood that a nsSNP will have a functional impact on the protein. It calculates the length of time (in millions of years) for a given amino acid that has been preserved to find the lineage leading to the protein of interest, and the longer the preservation time, the greater the likelihood of functional impact. PANTHER-PSEP is the name of the method (position-specific evolutionary preservation) (H. Tang and Thomas, 2016).

#### 2.1.3 Structure-based methods

The I-Mutant is used to predict the stability changes upon mutations (Capriotti et al., 2005). It is based on support vector machine (SVM) and predicts the changes in DDG values calculated from the Gibbs free energy value changes in kcal/mol (Capriotti et al., 2005).

DUET (http://biosig.unimelb.edu.au/duet/) uses an integrated computational approach to study the effect of mutation (nsSNP) on protein stability in humans and other genomes. DUET uses two complementary approaches, mutation Cutoff Scanning Matrix (mCSM) and Site-directed mutation (SDM), in a consensus prediction. It integrates both the scores using support vector machine (SVM). The combined value is in the form of the actual free energy value ΔΔG. The input for the DUET web server is the PDB structure file or 4-letter PDB code along with single point mutation or systematic mutation. This tool gives the DUET score and SDM and mCSM score in the result. mCSM and SDM2 were used to study the protein stability of the effect of mutation (Pires et al., 2014).

CUPSAT (Cologne University Protein Stability Analysis Tool) (http://cupsat.tu-bs.de/) is a web server to predicts impact of point mutations on protein stability. The model can distinguish between secondary structure specificity and amino acid environment based on its solvent accessibility. The tools predict mutant stability by combining PDB structure with existing and new protein structures (Parthiban et al., 2006).

The STRUM web server (https://zhanglab.ccmb.med.umich.edu/STRUM/) predicts stability changes caused by non-synonymous single-point mutations (nsSNP). It uses a boosting algorithm to predict the fold stability change (G) of protein molecules when they are mutated (Quan et al., 2016).

#### 2.1.4 Pathogenicity based methods

PredictSNP (https://loschmidt.chemi.muni.cz/predictsnp/) is a Consensus classifiers tool for predicting disease-related mutations. It provides easy access to other tools for the consensus classifier PredictSNP as well as annotations from the Protein Mutant Database and the UniProt database. To predict pathogenicity, it uses data in fasta format. Other measures, such as MAPP, PHD-SNP, and others, are also available through this server (Bendl et al., 2014).

REVEL (rare exome variant ensemble learner) is an ensemble method for predicting the pathogenicity of missense variants that is based on individual tools such as MutPred, FATHMM, PolyPhen, VEST, SIFT, MutationAssessor, PROVEAN, MutationTaster, LRT, SiPhy, GERP, phyloP, and phastCons. To identify pathogenic variants among a list of rare variants, pre-computed REVEL scores were provided for all possible human missense variants. The REVEL score assigned to a single missense variant can range from 0 to 1. Higher scores indicate that the variant is more likely to be pathogenic (Ioannidis et al., 2016).

#### 2.1.5 Consurf evolutionary conservation analysis

The consurf web server (http://consurf.tau.ac.il) is a tool for analysing the evolutionary pattern of macromolecule amino acids or nucleic acids to determine the region important for structure and function. ConSurf web server analyses sequence or structure input and gathers homologs, infers multiple sequence alignment and reconstructs a phylogenetic tree that reflects their evolutionary relationship. The alignment can be done with MAFFT-L-INS-I, PRANK, Muscle, or Clustalw. The conservation scores for each residue were calculated using Bayesian methods or Max. Likelihood (ML). Consurf prediction scores range from 1 to 9, with 1 indicating a variable region, 5 indicating an intermediate conserved region, and 9 indicating a highly conserved region (Ashkenazy, 2016).

### 2.2 Sequence retrieval and analysis

The UniProt database was used to retrieve the protein sequence of the human CASR gene (UniProt ID: P41180; 1,078 amino acid). Multiple sequence alignment was performed for WT and mutant protein sequences using multiple sequence viewer panels of Schrodinger’s maestro.

### 2.3 Retrieval of 3D protein structure and modeling

At the time of this study, the CaSR ECD PDB crystal structures were 5FBH, 5FBK, 5K5T, and 5K5S. This further investigation made use of a crystal structure without mutation (PDB ID: 5FBK, with a resolution of 2.10 Å) bound with Mg^2+^ and co-agonist tryptophan derivatives (Supplementary Figure S3). The Swiss model (Waterhouse et al., 2018) was used to generate a homology model for a missing loop based on the PDB template (PDB ID: 5FBK A). The SwissModel PDB was then used to obtain 3D protein structure coordinates for the missing loop region (amino acids Asn336-Asp377). For each mutation, mutated models were generated using Schrodinger’s Maestro. Protein preparation was performed on all WT and mutant protein structures using Schrodinger’s protein preparation wizard (Madhavi Sastry et al., 2013). The protein preparation step includes the following steps: The protein was pre-processed for bond order assignment, the addition of hydrogens, the formation of zero-order bonds to metals, the formation of disulfide bonds, the filling of missing loops and missing residues with Prime, the removal of water, and the capping of the terminal. The protein structure was refined by optimising the h-bond network with PROPKA at pH 7.4 and minimization with Banks et al. (2005) force fields (Jorgensen and Tirado-Rives, 1988).

### 2.4 Molecular dynamics simulation

Desmond (version 2020–1) was used to simulate the molecular dynamics of the WT and mutant proteins. The TIP3P solvation model was used to create the systems for molecular dynamics simulation. For each system, periodic boundary conditions were defined using a cubic box shape and the buffer method with a distance of 10 Å. Ions were strategically placed to neutralise the system. The Banks et al. (2005) force field was used to create the systems for molecular dynamics simulations.

For each system, the molecular dynamic simulation was run for 500 ns in triplicate. The energy was recorded every 1.2 ps, and the trajectory was saved every 100 ps. The volume of the box was equilibrated with the NPT ensemble at 300 K and 1.01325 bar pressure. Before selecting the simulation option, the systems were equilibrated using Desmond’s default relaxation protocol, i.e., Relax model system. The molecular dynamics simulation was performed with a 2 (fs) time step, Noose-Hoover chain temperature coupling (relaxation time 1.0 ps), and Martyna-Tobias-Klein pressure coupling (relaxation time 2.0 ps) of Isotropic style. The short-range method for coulombic interaction had a cutoff radius of 9.0.

#### 2.4.1 Analysis of MD trajectories

Desmond’s simulation event analysis programme was used to calculate various properties and measurements for all MD trajectory analyses. Root Mean Square Deviation (RMSD) provides information about the structural conformation of a protein throughout the simulation. It can indicate whether the simulation has equilibrated. The calculation of root mean square fluctuation (RMSF) assists in characterising the local changes or fluctuation around the amino acids in the protein chain. During MD simulation, the radius of gyration (Rg) indicates the compactness of the protein and its folding. Solvent Accessible Surface Area (SASA) is a key property of proteins that determines their folding and stability. Secondary Structure Elements (SSE) monitored throughout the simulation will aid in identifying the distribution of SSE by residue index throughout the protein’s structure. This analysis summarises the SSE composition for each trajectory frame over the course of the simulation. Hydrogen bonds (H-bonds) contribute to protein stability in a positive way. The number of hydrogen bonds in a protein indicates its stability. Salt bridges (SB) are involved in protein stability and folding, and changes in salt bridge contribution result in changes in protein stability.

### 2.5 Essential dynamics analysis

The essential dynamics analysis studies motions in the essential subspace, which has only a few degrees of freedom and is created by removing the overall translational and rotational motion because these are irrelevant for their internal motion. This returns atomic position expression values in a cartesian molecular coordinate system. This subspace describes the motions related to protein functions (Amadei et al., 1993).

The eigenvectors and eigenvalues were calculated using Schrodinger’s script “trj essential dynamics.py” with the default parameters, as well as the cross-correlation matrix plotted per-frame conformational deviations projected onto the calculated modes (principal component space). Eigenvectors (or principal components) represent positional deviations, and the magnitude of atomic fluctuations is associated with eigenvalues. As a result, the first principal component has the greatest positional deviation. The majority of the fluctuations are concentrated in a small subset of the few top PCs. Maestro and PyMol were used to visualise major protein motions. OriginPro 2021b was used to generate the PCA plot. In addition, the Porcupine plot was created in PyMol using the modevectors.py scripts. The Porcupine plot depicts motion directionality in 3D space, the geometry of 3D protein structure, and the magnitude of spikes, which represents motion strength.

## 3 Results

### 3.1 Prediction of single nucleotide variations impact using in-silico methods

The impact of non-synonymous SNVs was evaluated using various in-silico tools, and the results are shown in Table 1, which is a tabular record of SNV impact analysis. The results of SIFT, PROVEAN, and PHD-SNP revealed that all four mutations, P163R, I427S, D433H, and V477A, were predicted to be deleterious. Except for D433H, which was neutral, PredictSNP and MAPP analysis predicted that these mutations were deleterious. PolyPhen results >0.500 predicted that three mutations, P163R, D433H, and V477A, were possibly damaging, with PolyPhen scores of 0.906, 0.897, and 0.554, respectively. With the highest PolyPhen score of 0.996, mutation I427S was predicted to be probably damaging. CADD Phred score >20 indicates that all four mutations, P163R, I427S, D433H, and V477A, were the most deleterious, with the highest score of 27.7 for I427S. PON-P2 prediction revealed that mutations P163R, I427S, and D433H were pathogenic with a score value > -0.8, and mutation V477A result was unknown with a score of -0.65. PANTHER analysis revealed that all four mutations were probably damaging with a score of ≥0.57.

**TABLE 1:** Tabular record of in-silico method analysis result to predict the impact of SNVs over structure and functions.

S. no.	1	2	3	4
mutTCPdb ID	tcp8461	tcp8462	tcp8463	tcp8464
Mutation	P163R	I427S	D433H	V477A
Sift	Deleterious (0)	Deleterious (0)	Deleterious (0.01)	Deleterious (0)
PolyPhen	Possibly damaging (0.906)	Probably damaging (0.996)	Possibly damaging (0.897)	Possibly damaging (0.554)
CADD PHRED	26	27.7	27	26
PROVEAN	Deleterious (−8.537)	Deleterious (−4.878)	Deleterious (−3.258)	Deleterious (−3.377)
PON-P2	Pathogenic (−0.883)	Pathogenic (−0.883)	Pathogenic (−0.819)	Unknown (−0.65)
PANTHER	Probably_ damaging (0.57)	Probably_ damaging (0.57)	Probably_ damaging (0.57)	Probably_ damaging (0.57)
I-Mutant (DDG kcal/mol)	Decrease (−1.30)	Decrease (−2.44)	Decrease (−0.32)	Decrease (−1.70)
DUET (DDG kcal/mol)	Destabilising (−1.668)	Destabilising (−3.704)	Destabilising (−0.637)	Destabilising (−2.1)
CUPSAT (DDG kcal/mol)	Destabilising (−3.37)	Destabilising (−7.85)	Destabilising (−1.63)	Destabilising (−5.05)
REVEL (expected accuracy)	Pathogenic (−0.958)	Pathogenic (−0.938)	Pathogenic (−0.736)	Pathogenic (−0.908)
PredictSNP (expected accuracy)	Deleterious (−0.869)	Deleterious (−0.655)	Neutral (−0.752)	Deleterious (−0.506)
MAPP (expected accuracy)	Deleterious (−0.783)	Deleterious (−0.589)	Neutral (−0.653)	Deleterious (−0.588)
PhD-SNP (expected accuracy)	Deleterious (−0.875)	Deleterious (−0.589)	Deleterious (−0.589)	Deleterious (−0.676)

The I-Mutant server predicted the differences in Gibbs free energy (DDG kcal/mol) between WT and mutant proteins. As a result, the mutation I427S had the greatest impact on structure stability, with a DDG value of -2.44 kcal/mol. The other three mutations also had an effect on structure, resulting in decreased stability with DDG values of -1.30, -0.32, and -1.70 kcal/mol for P163R, D433H, and V477A, respectively. As a result of the I-Mutant server prediction, all four mutations have decreased protein stability. Similarly, DUET and CUPSAT support the findings of I-mutant, indicating that these mutations have a significant impact on structure stability, as indicated by the DDG values in Table 1. In terms of DDG values, mutant I427S has the highest degree of stability changes. According to REVEL analysis, all four mutations were pathogenic.

#### 3.1.1 Evolutionary conservation analysis

**FIGURE 2:**
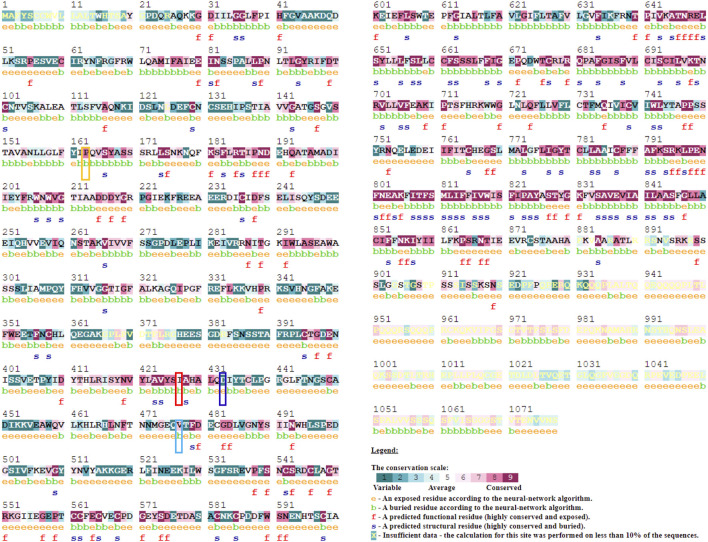
Evolutionary conservation analysis using Consurf webserver to predict the importance and impact of amino acid mutation at the respective site. A conservation scale depicted in figure ranging from score 1 (variable) to 9 (Conserved) with color variation. Mutation site highlighted with Yellow (P163R), Red (I427S), Blue (D433H), and Cyan (V477A) color.

In addition to these prediction methods and tools, consurf analysis results show the conservation of the amino acid at positions P163 (score 8), I427 (score 6), and V477 (score 7), except for D433 (score 1). This means that any changes to the amino acids at these positions will result in structural and functional changes to the protein. P163, I427, and V477 amino acids are found in the buried region. In contrast, consurf results show that amino acid D433 is present in the exposed region (Figure 2).

### 3.2 Protein sequence and structure analysis

Analysis of crystal structure (Supplementary Figures S4,S5) revealed that P163R amino acid mutation occurs in the coil region of LBD1, mutations I427S, and D433H occurs in the helix of hinge region, and mutation V477A occur in beta-sheet of hinge region. Overall, these four mutations occurs in CaSR ECD. . Cys60-Cys101, Cys358-Cys395, and Cys437-Cys449 were disulfide bridges in the 3-D structure, as shown in Supplementary Figure S5 using horizontal connections between amino acids. Supplementary Figure S4 depicts a magnified view of CaSR ECD that includes the metal ion binding site, co-agonist/ligand binding site, superimposition of mutant residues, and a dissected view of CaSR ECD. Supplementary Figures S4B,C) show binding site residues analysis for the initial PDB structure (PDB ID: 5FBK A) within a distance cutoff of 5.0 Å from the metal ion and co-agonist, respectively. The H-bond interactions measured for Mg^2+^ ion with amino acid residues were Ile81, Ser84, Leu87, and Leu88.

Ser147, Ser168, Ser169, Ser170, and Glu297 had similar interactions with co-agonist. Additionally, binding site residues within a defined cutoff of 5.0 Å were displayed. Superimposed residues at mutation sites were shown for both wild type (WT) and mutants in Supplementary Figures S4D,E depicts a dissected view of CaSR ECD to clearly distinguish the position of secondary structures in 3D structure.

Each amino acid has the physicochemical properties required for its structure and function. Thus, mutation of amino acids at specific positions results in a change in physicochemical properties. This change, however, may involve changes in size, charge, interactions, and other properties. In this case, the resultant residue increased in size, gained +1 charge, and decreased in hydrophobicity due to the mutation P163R. This mutation occurs in the protein’s ligand-binding domain 1 (LBD1), which may be involved in its functional activity. Changes at this position may affect the protein’s function. The D433H mutation causes an increase in size, and the WT residue is negatively charged, whereas the mutant is positively charged. In the case of V477A, there was a reduction in the size of the mutant residue, which may be involved in less interaction and thus be a cause of structural changes.

### 3.3 Molecular dynamics simulation study

The study of molecular dynamics simulation provides insight into the atomic details of protein structure and its dynamical behaviour when structure changes (Chitongo et al., 2020; Li et al., 2018; Mutt and Sowdhamini, 2016). The effect of gene SNVs associated with TCP on its protein structure and functions was determined using an MD simulation study for 500 ns in triplicate of each protein system (WT, P163R, I427S, D433H, and V477A). For each wild type and mutant, five protein systems were created (P163R, I427S, D433H, and V477A). MD simulation trajectories were examined for RMSD, RMSF, Secondary structure elements (SSE), the radius of gyration, hydrogen bonds, salt bridges, SASA, and protein-ligand interactions.

#### 3.3.1 Mutation induced changes in conformational stability

The RMSD analysis was used to examine the conformational changes of protein atoms in comparison to the reference frame, which provides an inside look at protein stability. The initial and final frames of the MD simulation were visualised and aligned using PyMol to calculate the RMSD for c-alpha atoms (Supplementary Figure S6). This demonstrates the structure’s deviation from its initial frame in 3D space.

In addition, all WT and mutant initial and end frames were aligned separately (Supplementary Figure S6). The RMSD of C-alpha atoms calculated with PyMOl for the initial and final frames. The average differences in RMSD between initial and final frames were 6.520Å (WT), 4.317Å (P163R), 6.512Å (I427S), 4.798Å (D433H), and 5.517Å (V477A).

**FIGURE 3:**
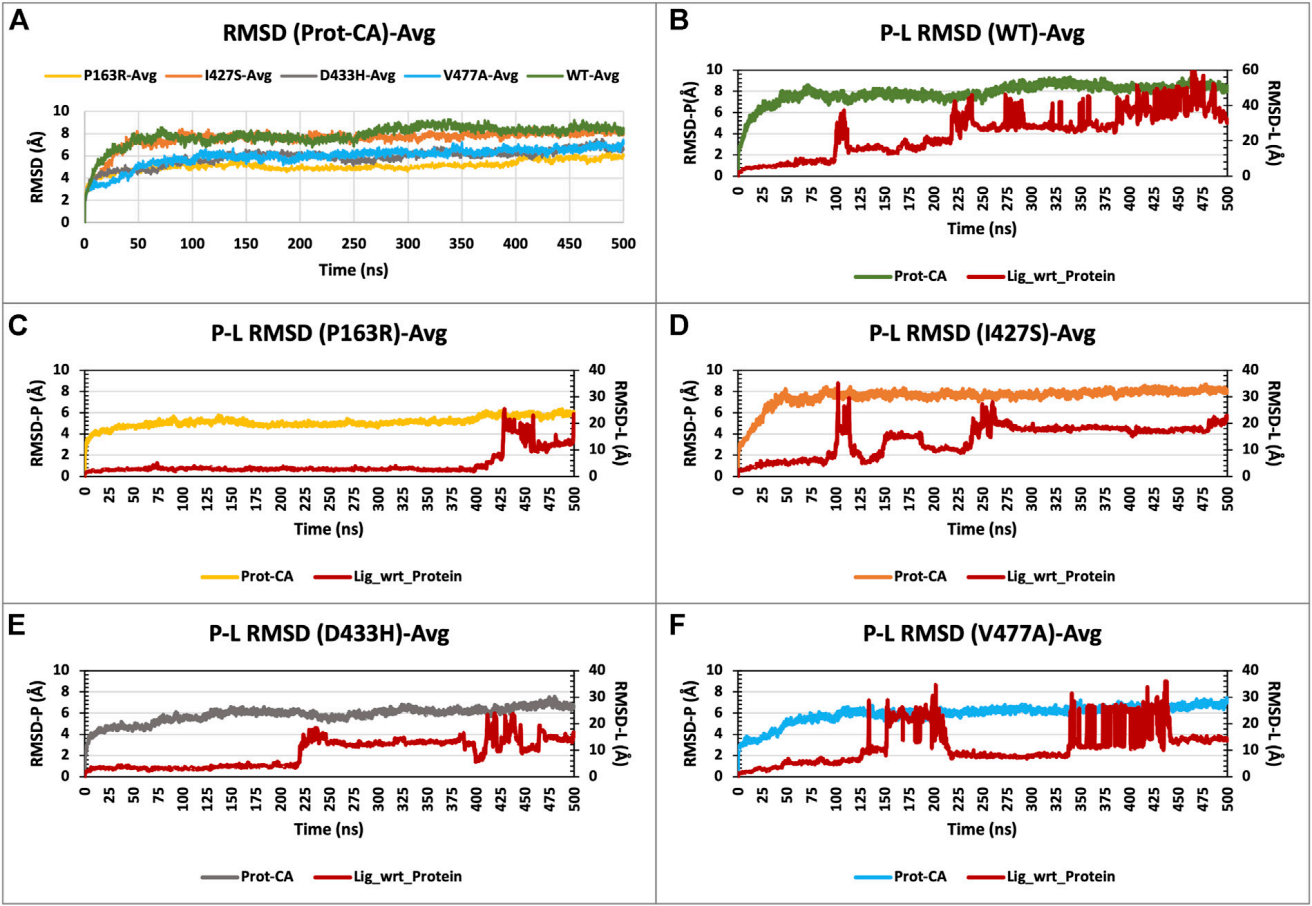
**(A)** RMSD plot of CaSR ECD protein C-alpha (Prot-CA) atoms throughout the simulation for WT and mutant proteins. **(B–F)** shows the RMSD plot for Protein-Ligand complex for WT, P163R, I427S, D433H, and V477A, respectively, throughout the simulation.

For each of the five systems, triplicate average RMSD plots for proteins and protein-ligand complexes were created (Figure 3 and Supplementary Figure S7). The Protein RMSD plot shows that the WT protein converged after 80 ns, P163R at 25 ns, I427S at 50 ns, D433S, and V477A at 100 ns. Throughout the simulation, the trajectories were analysed to determine the average and standard deviation in RMSD values (Figures 7A,B). This analysis depicts the average RMSD values for each system as follows: 7.842 Å (WT), 5.098 Å (P163R), 7.537 Å (I427S), 5.846 Å (D433H), and 5.936 Å (V477A). Standard deviation (SD) of RMSD indicates how far an RMSD deviates throughout the simulation. SD measures of RMSD were calculated to be 0.928 Å (WT), 0.479 Å (P163R), 0.905 Å (I427S), 0.734 Å (D433H), and 0.867 Å (V477A).

This analysis revealed that upon mutations, average and SD of RMSD values were decreased compared to WT protein, leading to conformational changes. Mutation P163R shows the higher dgree of changes in RMSD measurements.

RMSD plots of the Protein-Ligand complex (Figures 3B–F) revealed that ligand binding behaviour changed as a result of mutations. Ligand exhibited a distinct binding pattern with residues at the binding site; it remains intact for a specific period of simulation time before dissociating from its primary binding site. This protein-ligand RMSD plot shows comparable changes in RMSD and ligand binding behaviour as a result of mutations.

#### 3.3.2 Extended local fluctuation at mutation sites

**FIGURE 4:**
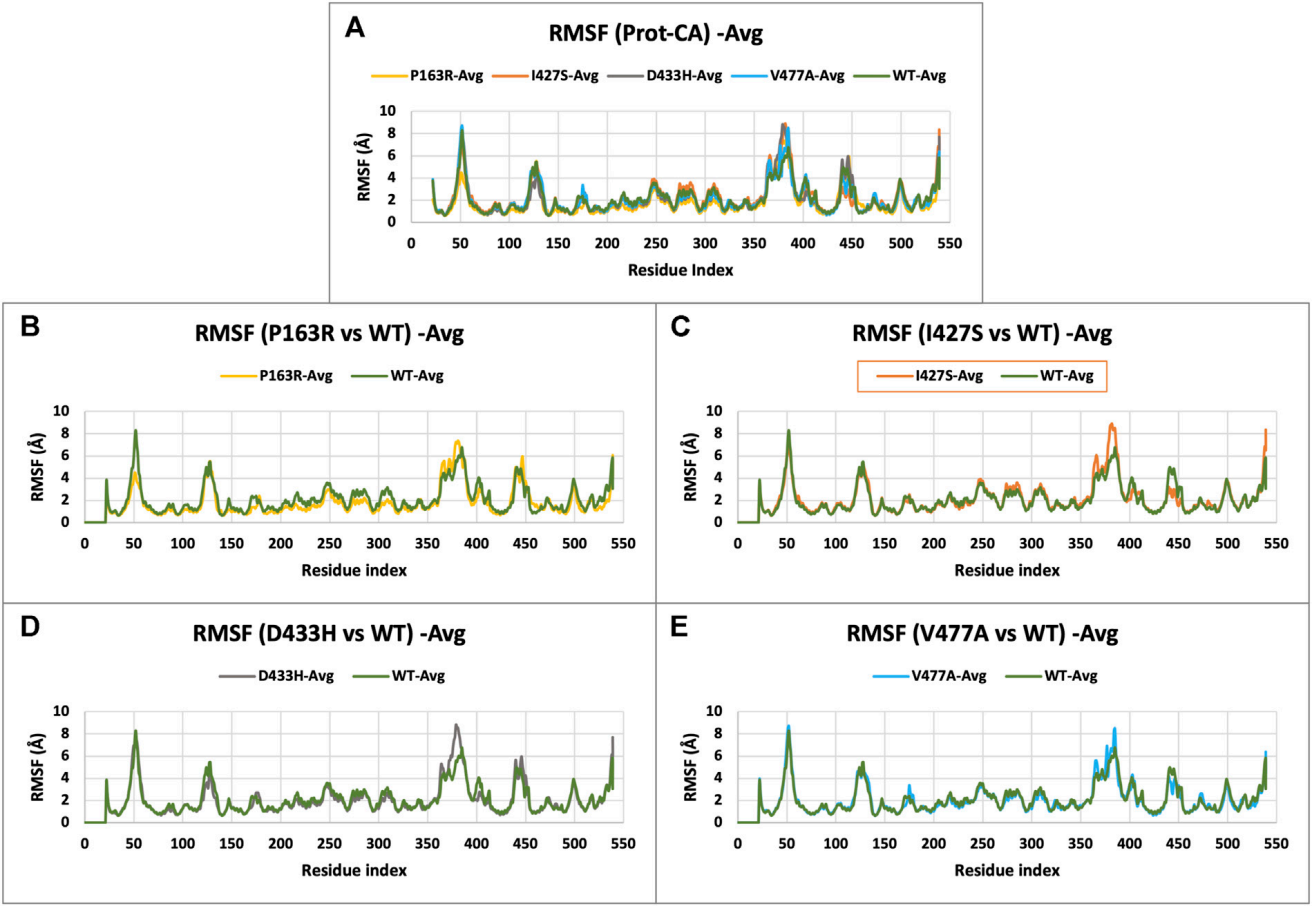
**(A)** RMSF plot of CaSR ECD protein C-alpha (Prot-CA) atoms throughout the simulation for WT and mutant proteins. **(B–E)** shows the RMSF plot for mutant P163R, I427S, D433H, and V477A, respectively.

The characterization of local changes along the protein chain is referred to as RMSF. It computes RMSF using the individual amino acid contribution. During the simulation, it aids in the analysis of fluctuating or stable amino acids. The protein’s terminal and loop regions are extremely mobile. The impact of a specific amino acid mutation on protein structure was investigated and compared using RMSF analysis from the trajectories of all five protein systems (Figure 4). The average RMSF values for WT and mutants P163R, I427S, D433H, and V477A, were 2.139 Å, 1.837 Å, 2.224 Å, 2.09 Å, and 2.1 Å respectively. The SD of RMSF were 1.256 Å, 1.281 Å, 1.429 Å, 1.469 Å, and 1.338 Å, respectively (Figures 7C,D). We also looked for the RMSF value at the mutation-specific position in each system and discovered that RMSF values increased upon mutation at mutation sites compared to WT (Table 2), indicating the deviation in fluctuations at mutation site. These findings imply that there was changed local flexibility in the protein structure and, as a result, decreased structural stability.

**TABLE 2:** Comparative average RMSF values (in angstrom) at mutation specific position throughout the simulation.

S. no.	Residue position	WT	P163R	I427S	D433H	V477A
1	P163	0.752	0.816	0.797	0.797	0.824
2	I427	0.825	0.809	0.854	0.725	0.686
3	D433	1.366	1.353	1.367	1.177	1.22
4	V477	1.489	1.196	1.557	1.464	1.422

#### 3.3.3 Variation in secondary structure elements upon mutation

Protein SSE were monitored throughout the simulation and summarised in the plot as a monitoring of each residue and its SSE assignment over time and SSE distribution by residue index throughout the protein structure (Supplementary Figures S9–S11). Table 3 summarises the percent SSE data values average out of triplicate. The distribution of SSE per residue explains how much the specific secondary structure is conserved during MD simulation. Protein SSE analysis revealed that the overall secondary structure distribution shows reduction of 1.32% (P163R), 1.43% (I427S), and 0.8% (D433H) % total SSE. While, mutation V477A shows slight (0.12%) increase in % total SSE. However, the distribution over residue index reveals a slight shift in SSE in relation to the amino acid position. In addition, the percent SSE plot (Supplementary Figure S9) revealed that in mutants P163R, I427S, and D433H, there was a loss of two beta-sheets near 370 residues, followed by the formation of an alpha-helix in mutant I427S around the same position. There was a depletion of alpha-helix in mutant P163R at 433 residue position, whereas two beta-sheets were present in mutant I427S for a small fragment of simulation time near residue 420. WT and mutant V477A SSE distributions differ significantly over time. This analysis revealed that the mutants 163R, I427S, and D433H exhibit a shift in secondary structure distribution throughout the simulation when compared to WT. Supplementary Figures S10,S11, also shows differences in positional SSE assignment over time.

**TABLE 3:** Average protein secondary structure elements throughout the simulation.

S. no.	System	% Helix	% Strand	% Total SSE
1	5FBK_A_WT	25.62	17.27	42.89
2	5FBK_A_P163R	24.6	16.97	41.57
3	5FBK_A_I427S	25	16.45	41.46
4	5FBK_A_D433H	25.51	16.57	42.09
5	5FBK_A_V477A	25.56	17.44	43.01

#### 3.3.4 Mutation associated behavior of unfolding

**FIGURE 5:**
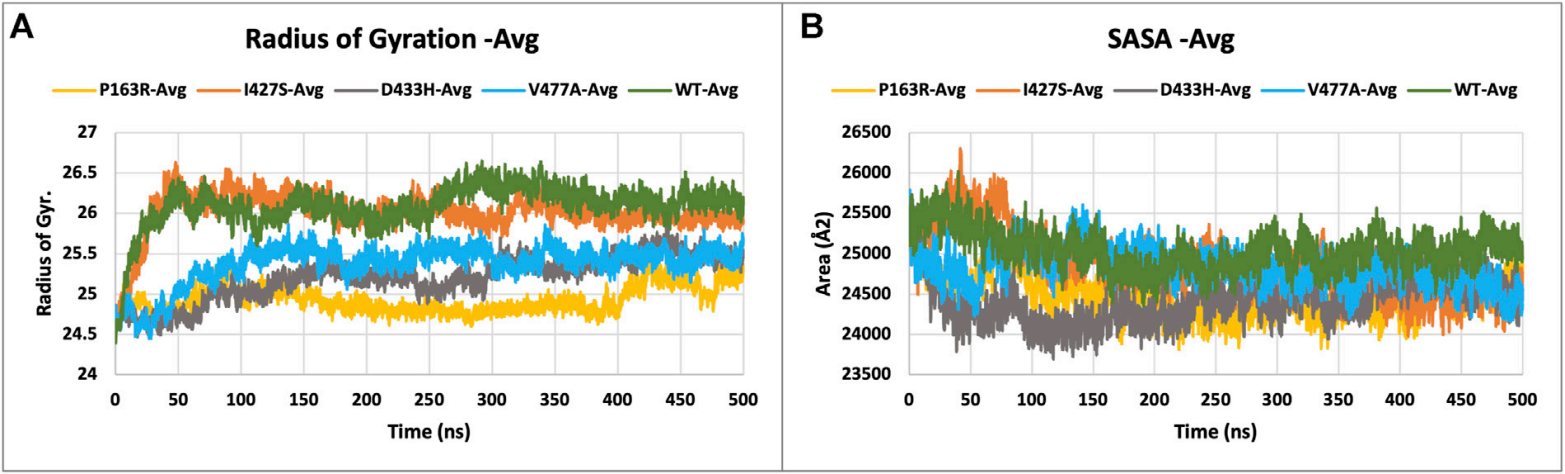
Plot **(A)** describing the radius of gyration (Rg), and **(B)** solvent accessible surface area (SASA), measurement of WT and mutant proteins throughout the simulation.

Protein compactness is measured using Rg values. When a protein maintains its fold stably, the value of Rg is relatively constant and low, and vice versa. It means that as a protein unfolds during the MD simulation, the Rg increases. The Rg data of WT and mutants were plotted and compared (Figure 5A). The average Rg values for WT, P163R, I427S, D433H, and V477A were 26.091 Å, 24.933 Å, 26.012 Å, 25.205 Å, and 25.372 respectively. Along with the average Rg value, the standard deviation in Rg values for WT, P163R, I427S, D433H, and V477A were 2.262 Å, 0.165 Å, 0.254 Å, 0.271 Å, and 0.249 Å,, respectively (Figures 7E,F). This analysis showed mutant P163R has significant deviation in Rg values.

#### 3.3.5 Solvent accessible surface area

The solvent accessible surface area (SASA) was measured to determine the effect of mutations on the folding or unfolding of the structure due to hydrophobicity changes. Hydrophobic residues are typically found in the protein’s core. The most advantageous space is the core due to solvent contact on the surrounding protein molecule. As a result, this phenomenon is important in protein packing or folding. The results of SASA analysis of WT and mutant (Figure 5B) show that mutation P163R and D433H causes a significant decrease in average SASA value. This behaviour causes the protein to fold tightly and to be more stable in folding and conformational changes. Other mutations, I427S, and V477A, have slight changes in SASA values when compared to a time series analysis. However, in terms of standard deviation, it was discovered that there is a significant deviation in SASA values throughout the simulation (Figures 7G,H). We concluded from this SASA analysis that mutation caused protein system instability when compared to the steady-state of WT protein.

#### 3.3.6 Hydrogen bonds and salt bridge analysis

**FIGURE 6:**
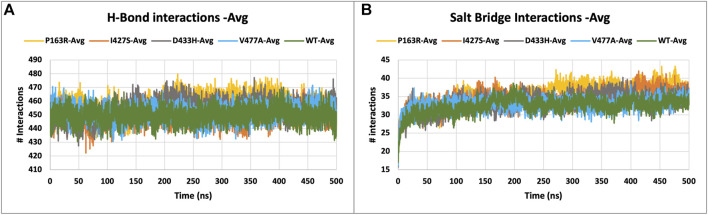
**(A)** H-bond interactions, and **(B)** Salt bridge interactions of WT and mutant proteins, measured throughout the simulation.

**FIGURE 7:**
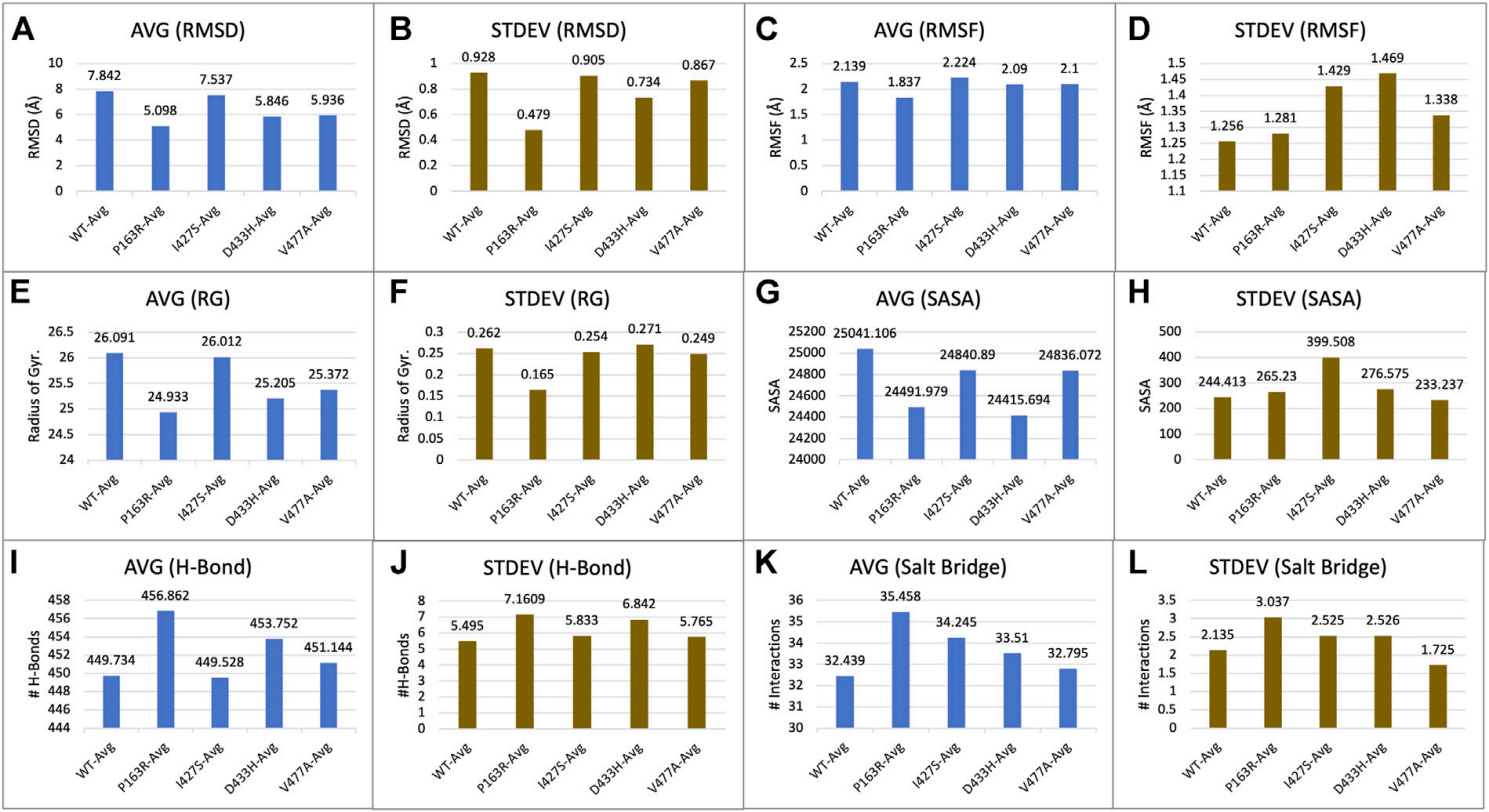
Statistical measure analyzed using MD simulation results. Plots showing triplicate average and standard deviations of RMSD, RMSF, Rg, SASA, H-bonds, and Salt bridges calculations for WT and mutants. **(A)** Average RMSD, **(B)** Standard deviation in RMSD, **(C)** Average RMSF, **(D)** Standard deviation in RMSF, **(E)** Average Rg, **(F)** Standard deviation in Rg, **(G)** Average SASA, **(H)** Standard deviation in SASA, **(I)** Average h-bond interactions, **(J)** Standard deviation h-bond interactions, **(K)** Average salt bridge interactions, **(L)** Standard deviation in salt bridge interactions.

Intramolecular hydrogen bonds (H-bonds) and salt bridges help proteins maintain their conformation. From MD simulation trajectories, we calculated the intramolecular H-bonds and salt-bridge of all proteins, WT, and mutants (P163R, I427S, D433H, and V477A). The graph in Figure 6 A shows the relationship between the number of hydrogen bonds and the time frame. This depicted the time series analysis of hydrogen bonds and depicted the significant changes in the number of hydrogen bonds over the specific time frame compared to WT. The average H-bonds of mutants P163R and D433H were higher than WT protein, while mutants I427S and V477A shows slight decrease in average H-bonds observed during the simulation. The standard deviation in H-bonds were incresaed upon mutations (Figures 6I,J). Throughout the simulation, the Salt bridge analysis plot revealed a difference in the number of salt bridge interactions between WT and mutant proteins (Figure 6B). Statistical measures of salt bridge analysis (Figures 7K,L) show that the average value of Salt bridge increased after mutation. However, when compared to WT, the Standard deviation increased after mutation.

#### 3.3.7 Protein-ligand interactions

A time-series analysis of protein-ligand interactions was performed using MD simulation trajectories, and a plot of amino acid residues interacting with the ligand was generated. The protein ligand interaction result depicts the interaction of a specific amino acid residue over the course of the simulation. In the case of multiple types of interactions possible by a residue, the interaction is considered a percentage (percent) value, which may be greater than 100 percent. Supplementary Figure S12 depicts ligand atoms in contact with amino acid residues for at least 30% of the simulation time. This interaction analysis could point to the effect of mutation on ligand-binding behaviour in the binding pocket. Tryptophan derivative is bound to the CaSR crystal structure at the orthosteric binding pocket between LBD1 and LBD2 of the CaSR (Figure 1). PL contact plot (Supplementary Figure S12) generated for the interactions made between protein and ligand during the simulation for more than 30% of simulation time, which showed the key amino acid residues for each protein. WT protein has no contact; mutant P163R has contacts with Ser147, Ser170, Ser171, and Glu297; mutant I427S has contacts with Asp190 and Glu297; mutant D433H has no contact; and mutant V477A has contacts with Asp190 and Glu297.

### 3.4 Essential dynamics analysis

In this study, essential dynamics analysis was performed using Schrodinger’s script, which was given the output of 10 PCs from MD simulation trajectories, as well as covariance data and a cross-correlation matrix for each WT and mutant protein P163R, I427S, D433H, and V477A. We measured the variance for each PC in WT and mutants (Supplementary Figure S13A) and found that mutants I427S, D433H, and V477A have higher variance than WT, except for mutant P163R.

The percent motion and percent aggregate motion of each PC (Supplementary Figures S13B,C) revealed that PC1 and PC2 percent aggregate motion changed significantly upon mutation. while the total number of PCs showing percent aggregation motion for each system shown in Supplementary Figure S13C were 80.39 (WT), 76.19 (P163R), 86.02 (I427S), 85.32 (D433H), and 84.99. (V477A).

**FIGURE 8:**
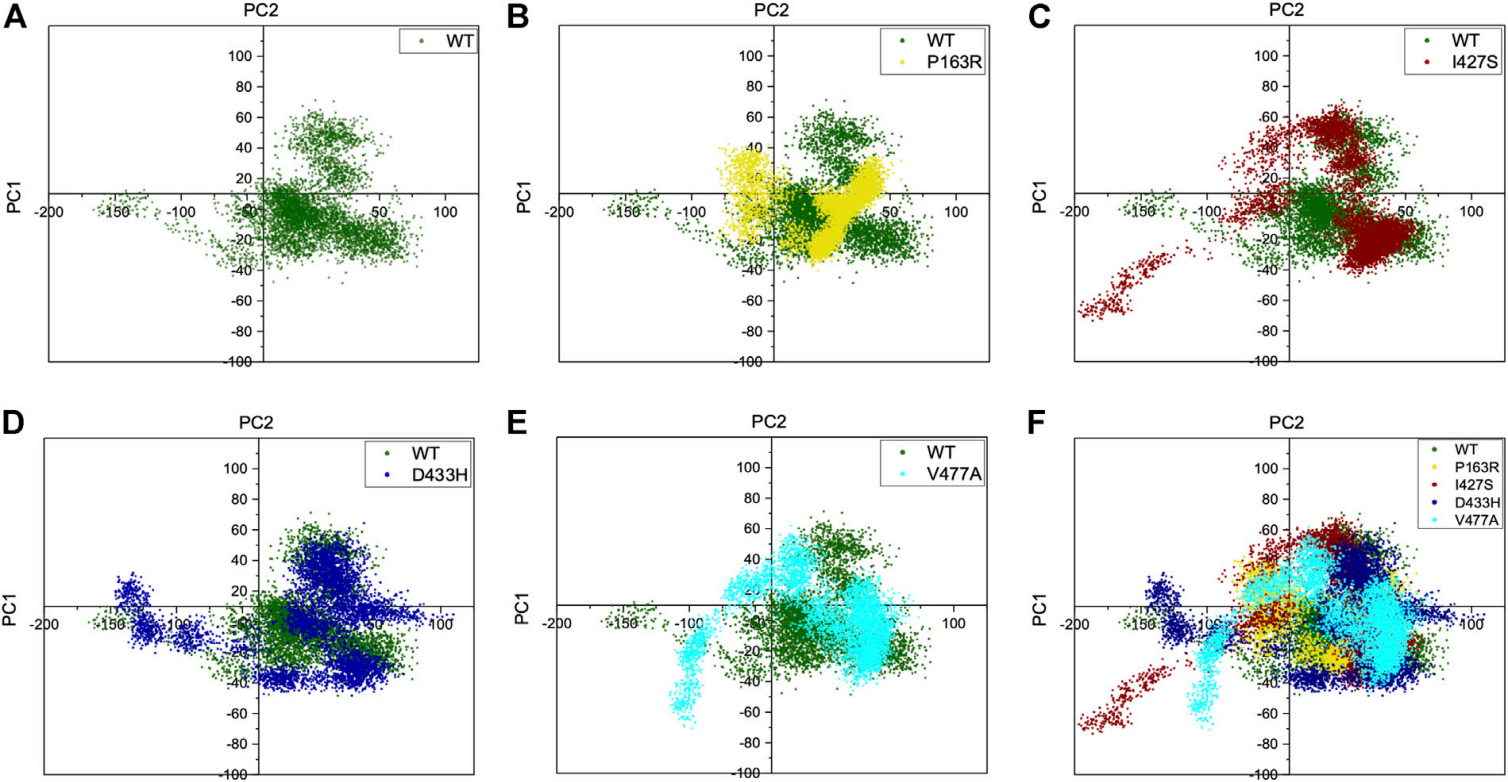
Projection of C-alpha atoms in essential subspace along with the first two principal components (PC1 and PC2) obtained from WT, P163R, I427S, D433H, and V477A. Figure **(A)** shows PC1 and PC2 of WT only. In contrast, figures **(B–E)** shows first two principal components of mutants, projected over WT principal components. **(F)** shows an overlay of WT and all four mutants PC1 and PC2 projection.

Furthermore, the top two PCs (PC1 and PC2) from each WT and mutant P163R, I427S, D433H, and V477A were analysed because these PCs account for the majority of protein motion during simulation. PC1 and PC2 were plotted on the *X* and *Y* axes, respectively, and the results were compared (Figure 8). This analysis revealed that mutants P163R, D433H, I427S, and V477A had significantly different collective motions of PCs than WT. This suggests that there was a change in protein dynamics, which may have resulted in altered protein structure and function.

#### 3.4.1 Cross-correlated motion of amino acid residues

**FIGURE 9:**
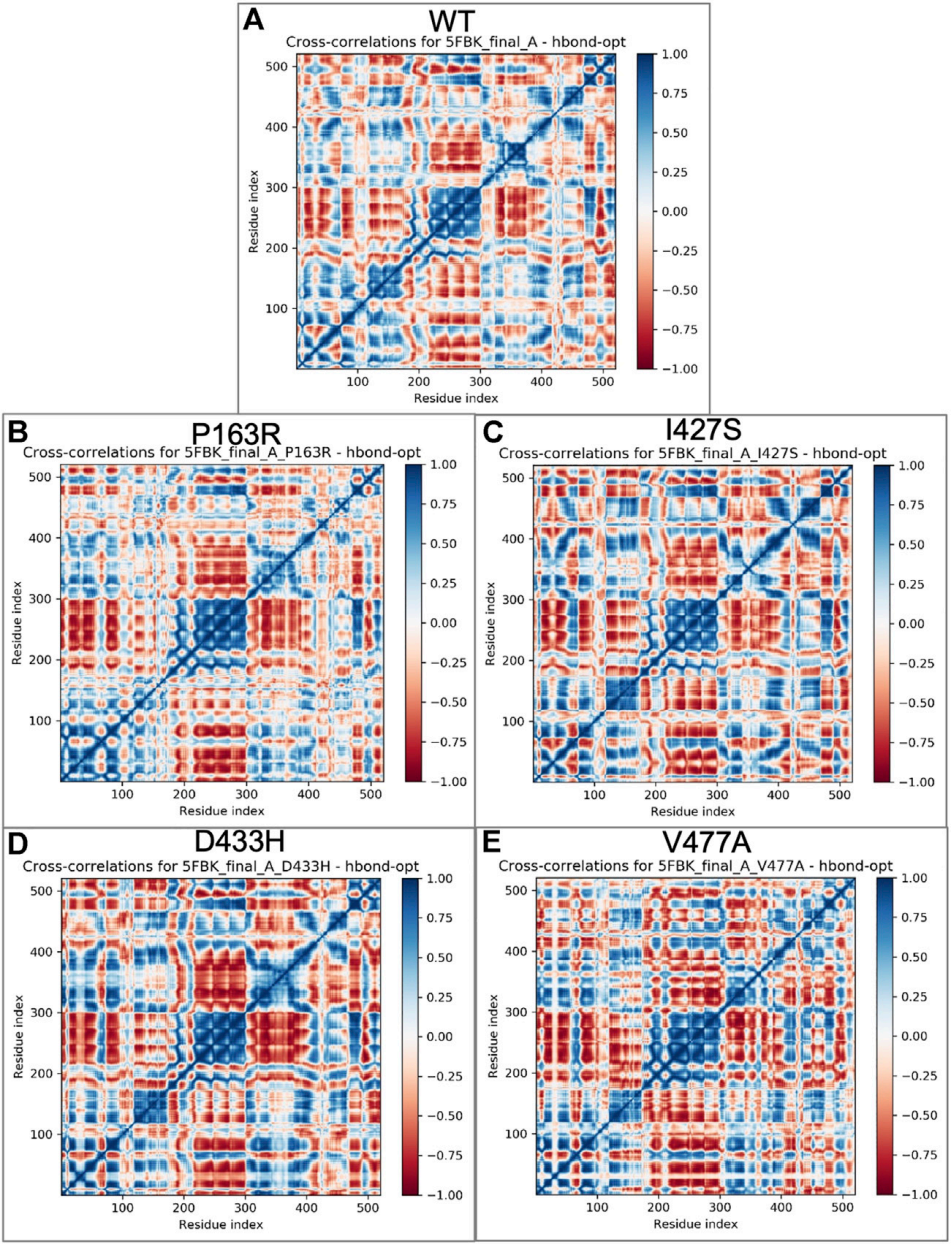
Cross correlations matrix obtained from essential dynamics of MD simulation trajectories for individual amino acid of **(A)** WT, **(B)** P163R, **(C)** I427S, **(D)** D433H, and **(E)** V477A, respectively. The color gradient represents correlated motion, where blue indicates highly correlated motion, and red indicates negatively correlated motion.

Cross-correlation analysis is useful for studying the dynamic behaviour of each amino acid in a protein chain in relation to other amino acids. For this, a cross-correlation matrix was generated for WT and mutant proteins using Schrodinger’s script (trj essential dynamics.py) (Figure 9). We observed significant changes in corelated motion as a result of mutation.

##### 3.4.2 Deviations in normal dynamics and protein motion

**FIGURE 10:**
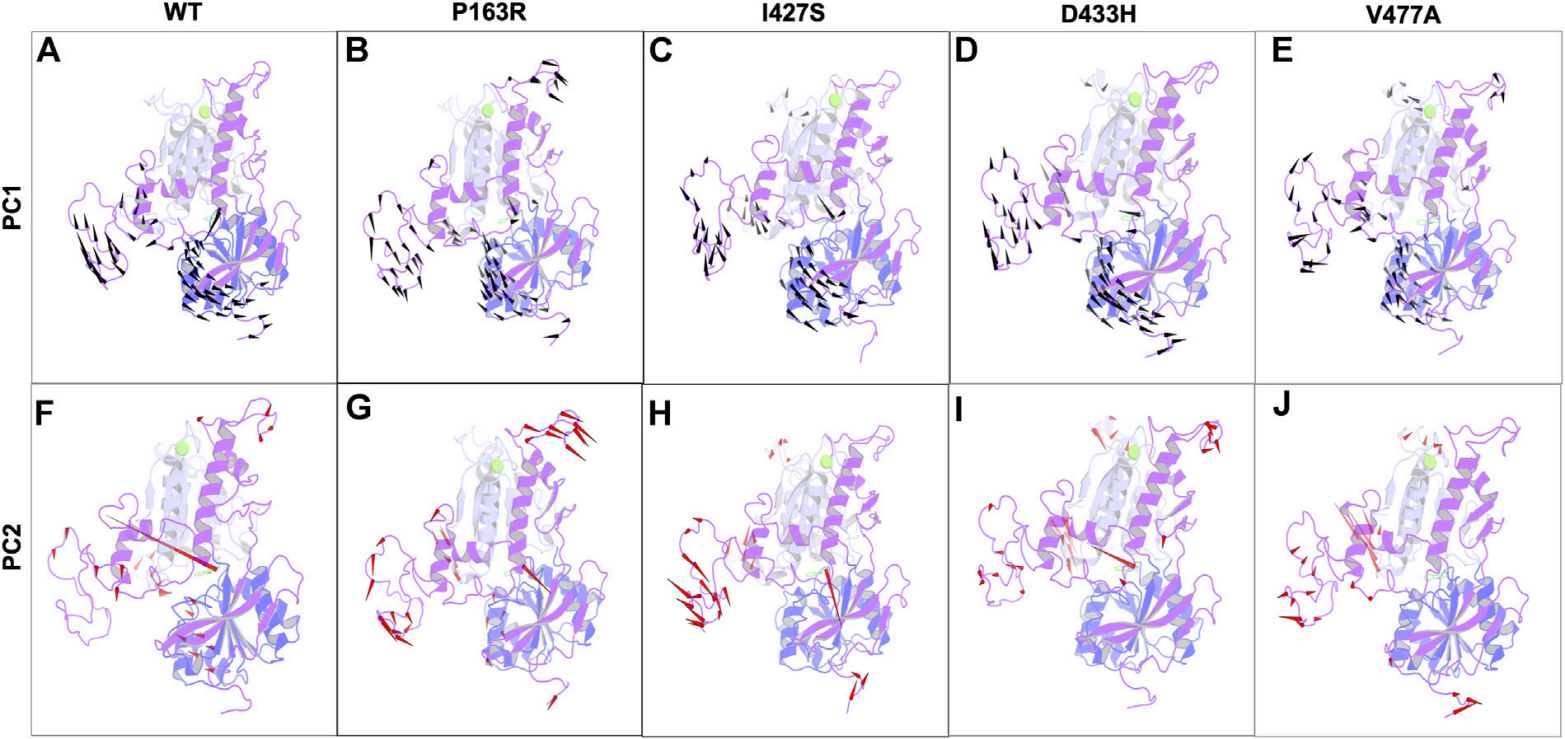
Porcupine plots for the first two principal components from essential dynamics of WT, and mutant P163R, I427S, D433H, and V477A MD simulation trajectories. Spikes for PC1 (Black) and PC2 (Red) originate from C-alpha atoms and represent the directionality of motions. The magnitude of spikes indicates the strength of motion.

In addition, for the first two major components, we generated the porcupine plot for WT and mutant proteins. The spikes originating from CA atoms in protein represent motion directionality with magnitude (Figure 10).

In-depth analysis of the porcupine plot of WT PC1 (Figure 10A) revealed that motion occurs in loops of LBD1, LBD2, and the hinge region, followed by the helix of LBD2 and beta sheets of the hinge region. The overall direction of motion was in different directions, indicating the expansion of space between domains. Simultaneously, the ligand was seen moving toward LBD2. The porcupine plot of WT PC2 (Figure 10F) has less motion than PC1. Motion occurs primarily in the loops of LBD1, LBD2, and the hinge region, as seen in the figure, as well as the helix of LBD2. In addition, ligand motion toward LBD1 was observed. As previously stated, the overall motion of mutant protein differed from that of WT protein, as shown in Figure 10. We concluded from this analysis of correlated motion and dynamics using a porcupine plot that the mutations P163R, I427S, D433H, and V477A cause deviations in normal dynamics and protein motion when compared to WT. Furthermore, there were significant changes observed from both PC for ligand dynamics upon mutation. These changes could affect protein function and dynamics, as well as ligand binding behaviour.

## 4 Discussion

TCP is a juvenile form of non-alcoholic chronic pancreatitis, exclusively seen in tropical countries. It is associated with dense intraductal calculi, severe abdominal pain, and further progress to diabetes (Barman et al., 2003). TCP patients has high risk to develop pancreatic cancer at later stage in lack of early detection and treatment (Chari et al., 1994; Barman et al., 2003). TCP is an idiopathic illness, and it remain unknown. Several gene SNVs have been identified in TCP patients and a list of gene SNVs obtained from literature survey reported in mutTCPdb database (Singh et al., 2018). Among all, CASR gene SNVs identified as one of the risk factors in TCP patients (Murugaian et al., 2008). CASR gene encodes for a class-C GPCR protein Calcium sensing receptor (CaSR), which has a significant role in pancreatic physiology. The CASR gene is highly expressed in the parathyroid gland and renal tubules of the kidney as well as the pancreas, islets of Langerhans, intestine, skin, brain, heart, bone, lung, and other tissues show expression (Rácz et al., 2002; Pidasheva et al., 2004; Hendy and Canaff, 2016). CaSR involved in monitoring and regulation of Ca^2+^ concentration in pancreatic juice by triggering ductal electrolytes and fluid secretion. Hypercalcemia raises the risk of acute pancreatitis by causing early activation of trypsinogen into trypsin, resulting in autodigestion of pancreatic parenchyma (Murugaian et al., 2008). Activation of CaSR results in increased HCO_3−_ secretion in the pancreatic ducts. Thereby reducing the Ca^2+^ salt precipitation in duct lumen and decreasing the risk of stone formation and pancreatitis (Rácz et al., 2002). CaSR associated with several disorders like familial hypocalciuric hypercalcemia (FHH), Neonatal severe hyperparathyroidism (NSHPT), Autosomal dominant hypocalcemia (ADH), Autoimmune hypocalciuric hypercalcemia (AHH), and Acquired hypoparathyroidism (AH). There were more than 200 mutations of CaSR have been identified and categorized into inactivating (FHH/NSHPT type) and activating (ADH type) mutations (Hannan and Thakker, 2013). The CASR gene may also be implicated in carcinogenesis, notably in the colon, breasts, and prostate, as well as cardiovascular and inflammatory illnesses, including both digestive and respiratory ailments (Vahe et al., 2017).

Various genetic and environmental influences have been described as causative factors for idiopathic pancreatitis. SPINK 1 and CFTR gene have been found to be associated with tropical calcific pancreatitis from various parts of the world. Multiple reports from Kerala and rest of India have identified SPINK 1, CFTR, CTRC, and MORC4/CLD locus gene mutations. Some studies have identified SPINK 1 mutations in up to 40% of patients with idiopathic pancreatitis. The identifications of these mutations have opened a new corridor in understanding the etiopathogenesis of pancreatitis. However, lack of GWAS studies on TCP may be the reason why it is still an unsolved mystery.

Mutation of CASR gene alone or in combination with SPINK1 gene mutation can lead to pancreatitis. Muruganian et al., in 2008 identified four novel CASR gene mutations in TCP patients, which shows that CASR gene variants alone cause idiopathic chronic pancreatitis. These four mutations (P163R, I427S, D433H, and V477A) were inactivating mutations and occur in Venus-flytrap domain of CaSR-ECD. A study also revealed that CaSR expression was decreased in the case of pancreatic cancer. Which suggests that activation of CaSR has some key role in pancreatic cancer (B. Tang et al., 2016).

In this study, we used in-silico techniques and the molecular dynamics approach to examine the influence of CASR gene SNVs on structure and function. In-silico tools and servers like SIFT, PolyPhen, CADD, PROVEAN, I-Mutant, PANTHER, PON-P2, Duet, CUPSAT, REVEL, PredictSNP, MAPP, PHD-SNP, and Consurf evolutionary analysis conferred the deleteriousness, destabilising, and pathogenicity of mutations based on sequence, structure, and evolutionary conservation analysis. The in-silico SNV impact analysis algorithm gives the result that all four mutations (P163R, I427S, D433H, and V477A) were predicted to be deleterious on protein function. The Gibb’s free energy changes (DDG) upon mutation showed destabilizing effects on the protein structure. Evolutionary conservation analysis revealed that amino acid position of P163, I427, and V477 are conserved. This suggests that mutation at this location would result in structural and functional changes in the altered protein. Also, these mutations may lead to changes in physiochemical properties of overall protein and subsequently results in different structural and functional behavior.

Further investigation carried out at atomic-level changes in various physical and structural characteristics of proteins/amino acids using MD simulation study of WT and mutant protein. Thus, we were able to observe a variety of behavioural changes by computing the RMSD, RMSF, Rg, SSE, H-bonds, salt-bridges, SASA, and Protein-ligand interaction. The conformational changes between the WT and mutant proteins are visulised using a time-series analysis of the RMSD over the simulation (Figure 3). The RMSD plot for protein-ligand complexes depicts the variation in ligand binding behaviour as a function of binding site residues. RMSF calculations of each amino aid residue in the protein chain were used to study local measures of fluctuation throughout the simulation. The RMSF plot (Figure 4), which was created for WT and mutant proteins, aids in inferring significant changes in peaks indicating fluctuations. We observed an increase in RMSF value upon mutations compared to WT protein. The radius of gyration (Rg) is critical in determining protein stability and folding pattern. The unfolding of the protein results in a higher Rg value, whereas proper folding or compactness of the protein results in a lower Rg value. Rg values observed throughout the simulation, as well as a comparison plot (Figure 5A) of WT and mutant Rg values, show clear differences in protein compactness in all protein systems. Result shows the decrease in protein compactness and stability while increased unfolding of protein upon mutations except for mutation P163R. SASA measurements revealed that the mutation caused changes in the SASA value of the protein (Figure 6B) when compared to the WT. SASA’s average value decreased as a result of the mutation. Among all mutations, P163R and D433H shows higher reduction in SASA. This variation in SASA could significantly alter protein folding and packing, resulting in altered protein function and normal behaviour. Several interactions contribute to thermodynamic stability and aid in protein folding. Interactions such as H-bond and salt bridge are important partners in this protein’s thermodynamic stability. For such stability, a collective measure of individual bond could be extremely valuable. Bonds are essential for the formation of secondary structures in proteins. These all changes could impact the protein’s mechanism to achieve its function and signalling. Additionally, ligand binding at binding pocket was also deviated in mutant protein in comparison to native protein, which will also affect the binding of small molecule and its subsequent therapeutic effect.

Further, essential dynamics analysis of concerted motion using MD simulation trajectories revealed the significant changes in dynamic behavior of protein. The major dynamic motions of protein from MD simulation trajectories were studied using principal component analysis and cross-correlation dynamics of concerted motion. This provides additional measures for studying the only motion that has a significant impact on protein function and stability. We discovered an increase in percent aggregate motion for the first two PCs after mutation, with I427S being the most affected, followed by D433H, V477A, and P163R. (Supplementary Figure S13). When comparing P163R to WT, the overall percent aggregate motions for all Top 10 Pcs were reduced. However, this mutation P163R is still a significant cause of changes in protein normal motion and dynamic behaviour. It was also discovered that due to mutation, there is a significant deviation in the projection of the first two PCs (Figure 8). Mutations P163R, I427S, and V477A clearly showed a difference in PC1 and PC2 projection over both PCs of WT, whereas mutation D433H follows a somewhat similar pattern in PC data but still showed significant differences compared to WT. Finally, there were significant differences in the PCs of mutant proteins compared to WT. The cross-correlation matrix, in addition to this motion study, suggests major changes in local fluctuations of protein residues, either positive or negative correlated motions. Overall, this study revealed that these mutations have significant impact on protein structure and subsequently affect the protein’s native function. This study may further utilize to understand the pathophysiology upon structural and functional changes of protein.

## 5 Conclusion

The CASR gene plays a significant role in the various physiological processes of food digestion, regulation of pancreatic secretions, homeostasis role of Ca^2+^ regulation, and others. Mutations in the CASR gene associated with TCP are still poorly untreated. Mutation in the CASR gene alone or combination with the SPINK1 gene is involved in the cause of pancreatitis or pancreatic cancer. CaSR mutations P163R, I427S, D433H, and V477A, are associated with TCP and reported in the mutTCPdb database. Several computational tools and methods were utilized in this study to see the impact of SNVs.

The results of this study indicate that the mutations P163R, I427S, D433H, and V477A have a significant impact on protein structure. Various measures such as deleteriousness, changes in Gibbs free energy (DDG) value, pathogenicity, and atomic-level analysis of various properties and measures using MD simulation were used. Changes in the amino acid at these specific positions, based on these observations, could harm its structural and functional properties. The evolutionary conservation analysis using the consurf server also suggests that the amino acid residues P163, I427, and V477 were buried and highly conserved. In contrast, amino acid residue D433 was exposed and slightly conserved.

MD simulation, along with essential dynamics analysis, also suggests that mutation P163R has a significant impact in all MD simulation measurements when compared to other mutations I427S, D433H, and V477A. However, in the end, all mutations have a clear impact on the structural and functional aspects of the protein. These amino acid changes are thus a compelling cause of altered protein functions and behaviours. Changes in co-agonist binding behaviour at the orthosteric site and interactions with amino acid residues can also be seen in the results. This study contributes to a better understanding of the impact of SNVs on structure and function, which may lead to the development of biomarkers and therapeutics.

## Data Availability

The original contributions presented in the study are included in the article/Supplementary Material, further inquiries can be directed to the corresponding authors.
