# Supplementary material for: Molecular dynamics study of tropical calcific pancreatitis (TCP) associated calcium-sensing receptor single nucleotide variation
Source: Front Mol Biosci. 2022 Oct 4;9:982831. doi: 10.3389/fmolb.2022.982831 (PMC9581290; doi:10.3389/fmolb.2022.982831)
Supplement: Supplementary file 1 [file DataSheet1.docx]

***Supplementary Material***

**Figures**


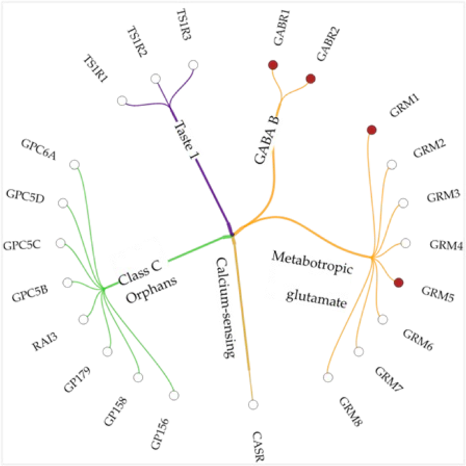


**Figure S1:** Classification of Class C-GPCR showing the CASR protein (Kooistra et al., 2021; Munk et al., 2016).

(Source of figure S1: GPCRdb (<https://gpcrdb.org/structure/statistics>) is licensed under CC BY 4.0 (https://creativecommons.org/licenses/by/4.0/) provided in the legal notice section of about GPCRdb documentation page (https://docs.gpcrdb.org/legal_notice.html).


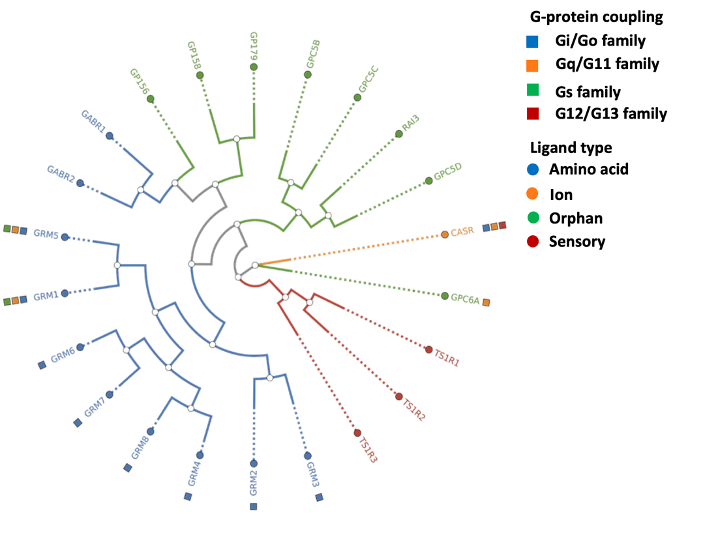


**Figure S2:** The phylogenetic tree of class C GPCRs shows each receptor's G-protein coupling and ligand types (Isberg et al., 2016; Kooistra et al., 2021).

(source of figure S2: GPCRdb (<https://gpcrdb.org/phylogenetic_trees/render_v3#>) is licensed under CC BY 4.0 (https://creativecommons.org/licenses/by/4.0/) provided in the legal notice section of about GPCRdb documentation page (https://docs.gpcrdb.org/legal_notice.html).


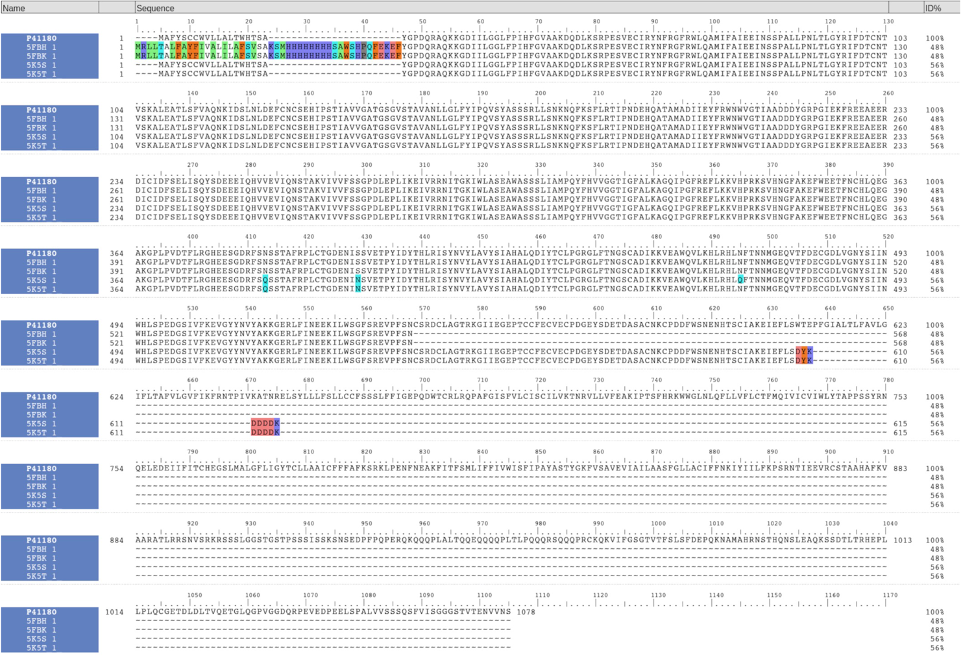


**Figure S3:** MSA of CaSR reference protein sequence (UniProt ID: P41180) with 3D structure available in PDB database (PDB ID: 5FBH, 5FBK, 5K5S, and 5K5T) shows a mutation in respective crystal structure concerning reference protein sequence.


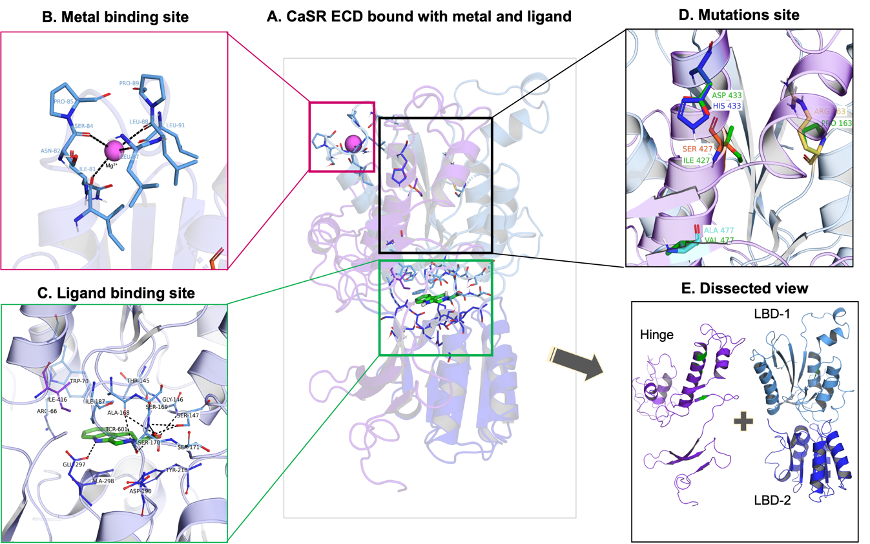


**Figure S4:** Shows the orientation of amino acid residues at mutation sites, metal, and ligand binding sites, along with interactions. (A) A monomer of CaSR ECD bound with metal ion Mg^2+^ and co-agonist L-tryptophan; (B) H-bond interactions between the metal ion and its binding site residues; (C) H-bond interactions between the co-agonist L-tryptophan and binding site residues; (D) Highlighted view of superimposed WT and mutant residues in 3D structures; and (E) A dissected view of the individual domain of CaSR ECD.


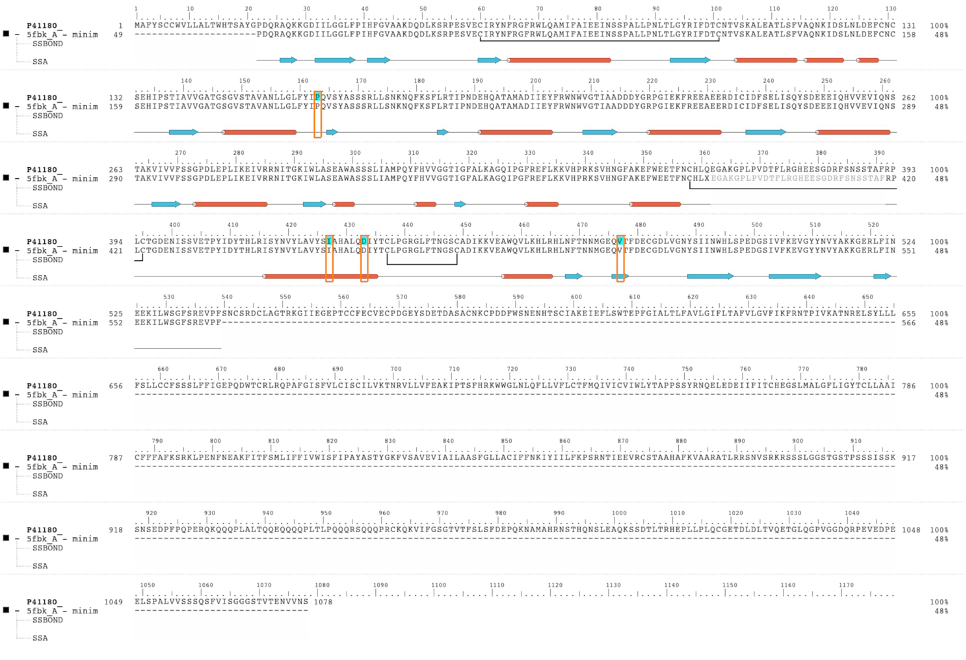


**Figure S5:** MSA of CaSR reference protein sequence (UniProt ID: P41180) with the crystal structure (PDB ID: 5FBK). The secondary structure assigned is shown in the figure based on the crystal structure. The mutation associated with TCP is highlighted with the respective amino acid position. The disulfide bridge between cysteine residues is represented by a horizontal line connecting the cysteine residues at the bottom of the sequence.


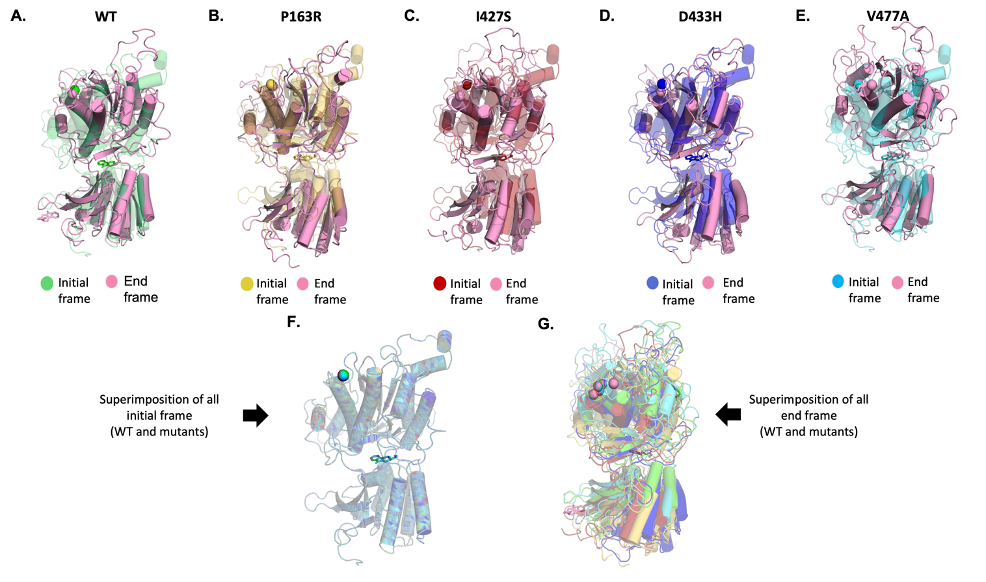


Figure S6: This shows the superimposition of initial and end frame structures obtained from MD simulation trajectories for (A) WT, (B) P163R, (C) I427S, (D) D433H, (E) V477A, protein, respectively. (F) depicts the superimposition of initial frames and (G) superimposition of end frames, obtained from WT, P163R, I427S, D433H, and V477A.


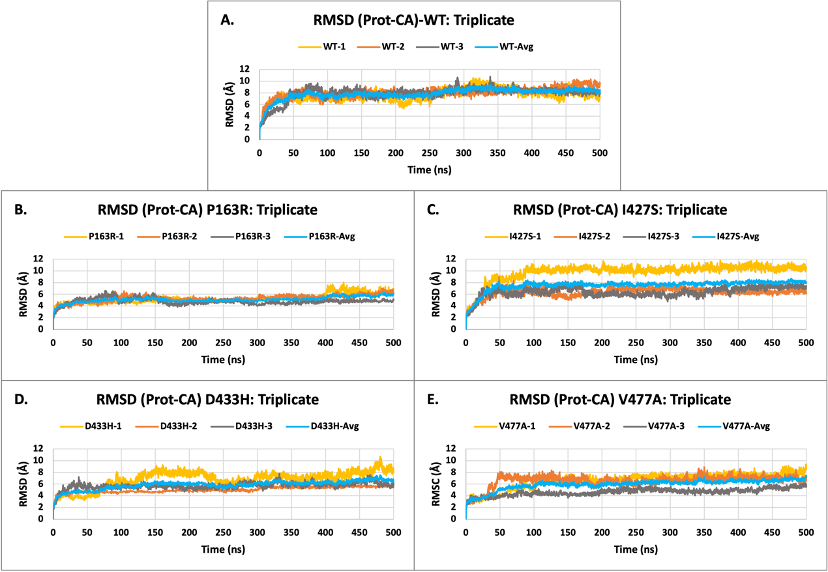


**Figure S7:** RMSD plot of CaSR ECD protein C-alpha (Prot-CA) atoms in triplicate MD run for WT and mutant proteins.


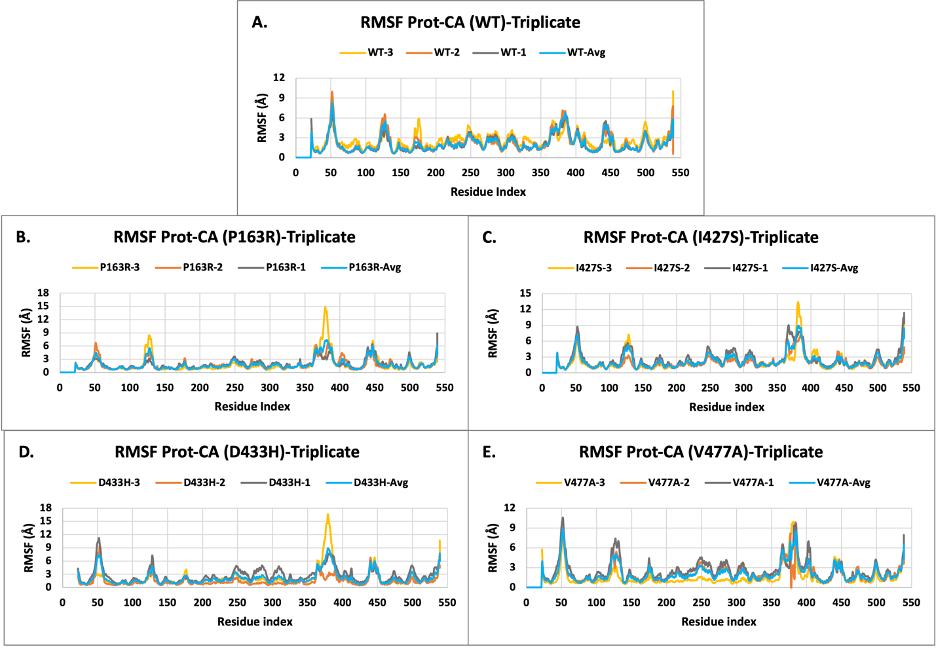


**Figure S8:** RMSF plot of CaSR ECD protein C-alpha (Prot-CA) atoms in triplicate MD run for WT and mutant proteins.


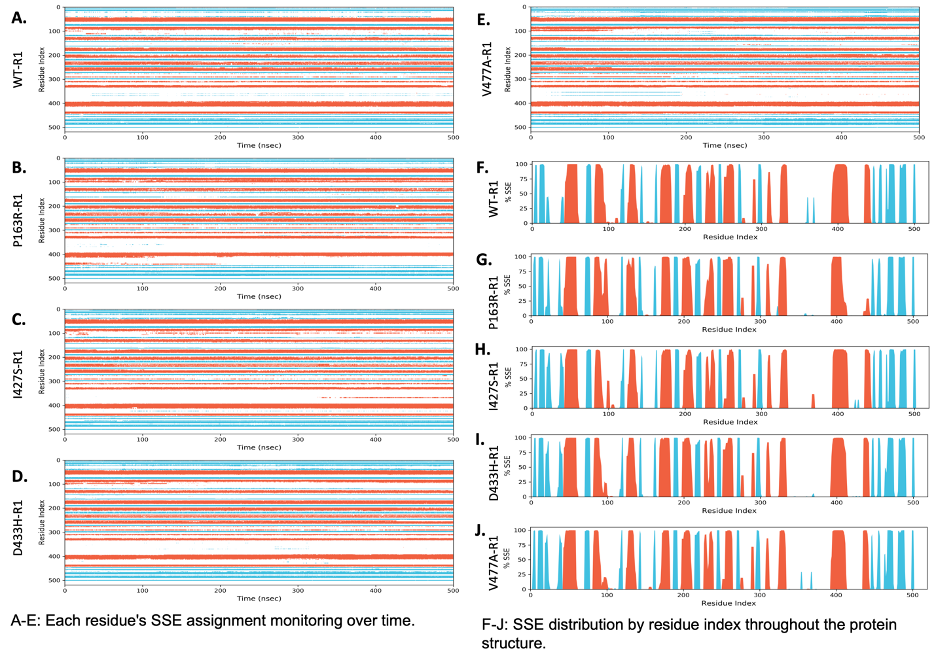


**Figure S9:** MD Run-1, secondary structure analysis. Plot (A-E) displays monitoring of each residue's Secondary structure elements (SSE) assignment over time for WT and mutant proteins. Plot (F-J), Shows SSE distribution by residue index throughout the protein structure calculated throughout the simulation time for WT and mutant proteins. Red color represents alpha-helix and cyan color represents beta-sheets.


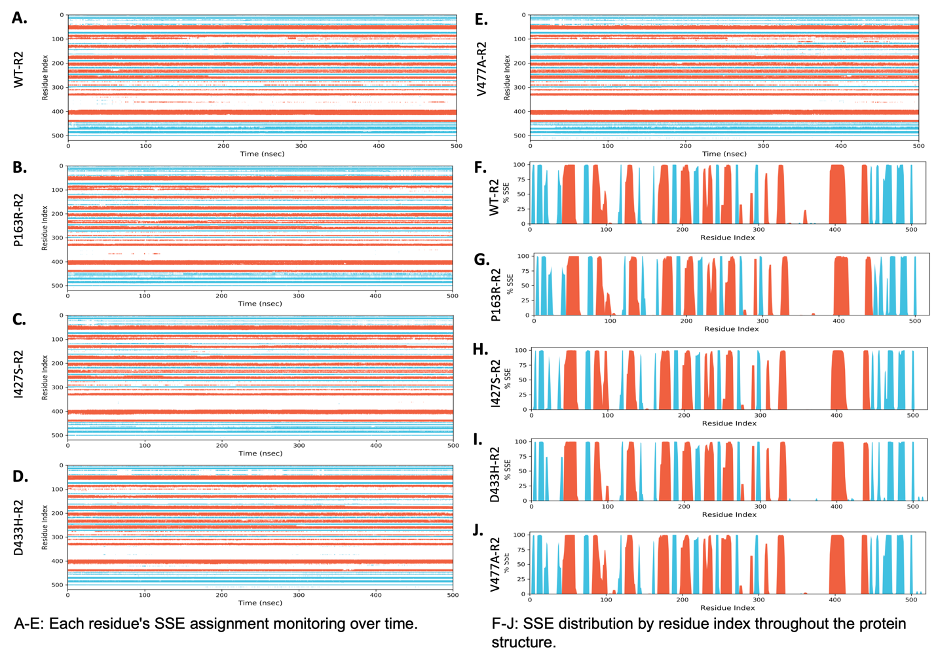


**Figure S10:** MD Run-2, secondary structure analysis. Plot (A-E) display monitoring of each residue's Secondary structure elements (SSE) assignment over time for WT and mutant proteins. Plot (F-J), Shows SSE distribution by residue index throughout the protein structure calculated throughout the simulation time for WT and mutant proteins. Red color represents alpha-helix and cyan color represents beta-sheets.


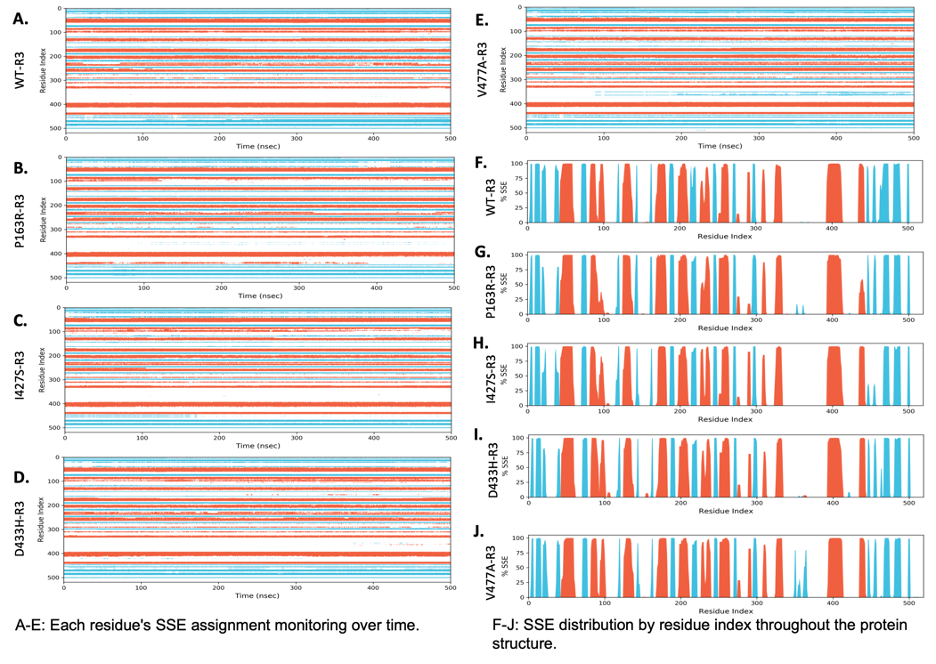


**Figure S11:** MD Run-3, secondary structure analysis. Plot (A-E) display monitoring of each residue's Secondary structure elements (SSE) assignment over time for WT and mutant proteins. Plot (F-J), Shows SSE distribution by residue index throughout the protein structure calculated throughout the simulation time for WT and mutant proteins. Red color represents alpha-helix and cyan color represents beta-sheets.


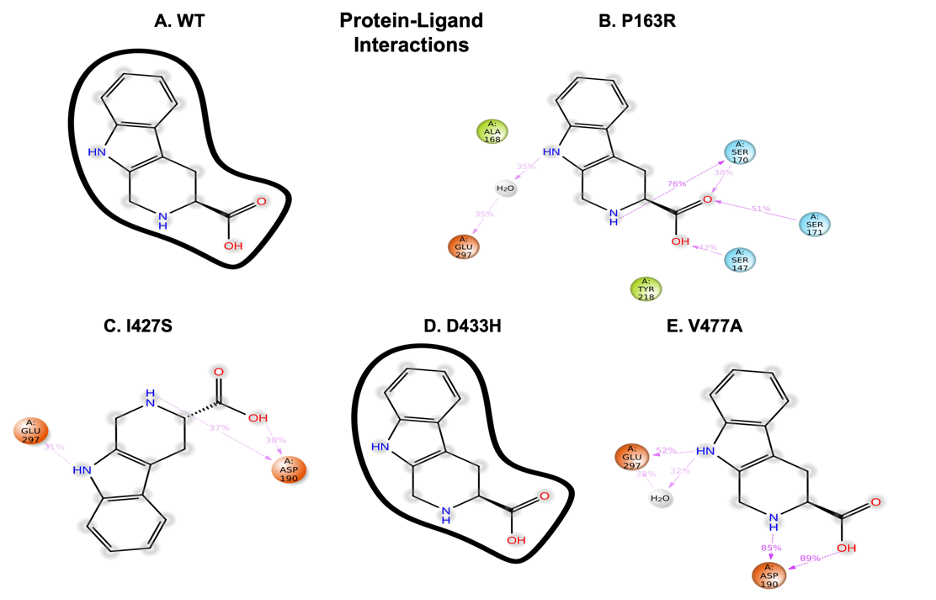


**Figure S12:** Schematic detailed ligand atoms interactions with protein residues, occurring more than 30.0% of the simulation time. (A) WT, (B) Mutant P163R, (C) Mutant I427S, (D) Mutant D433H, and (E) Mutant V477A. Orange color (Negative charged), cyan color (Polar), green color (Hydrophobic), and grey color shows the solvent exposure.


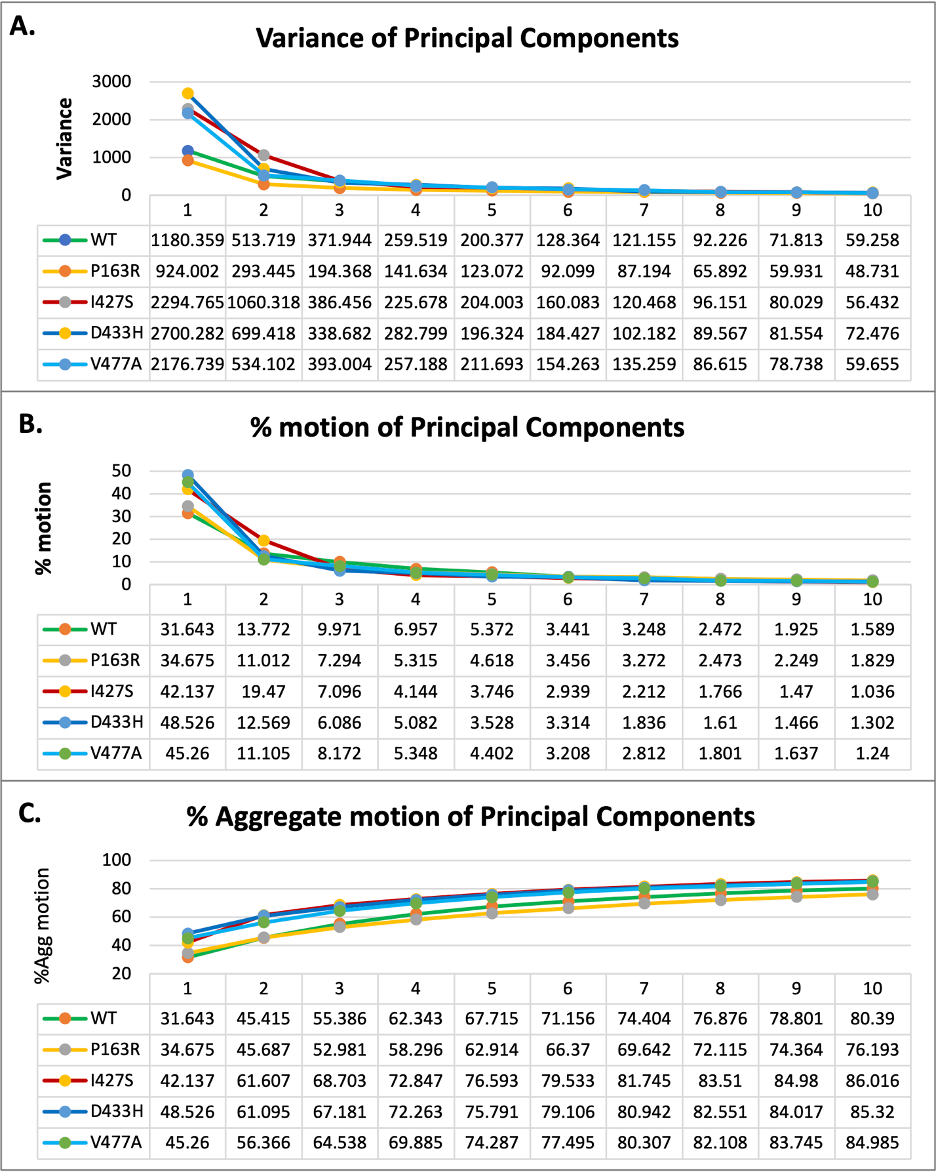


**Figure S13:** Plots of (A) variance, (B) %motion, and (C) % aggregate motion, for each Principal Components (PCs) along with tabular data of WT and mutants (P163R, I427S, D433H, and V477A).

**References:**

Isberg, V., Mordalski, S., Munk, C., Rataj, K., Harpsøe, K., Hauser, A. S., Vroling, B., Bojarski, A. J., Vriend, G., & Gloriam, D. E. (2016). GPCRdb: An information system for G protein-coupled receptors. *Nucleic Acids Research*, *44*(D1), D356–D364.

Kooistra, A. J., Mordalski, S., Pándy-Szekeres, G., Esguerra, M., Mamyrbekov, A., Munk, C., Keserű, G. M., & Gloriam, D. E. (2021). GPCRdb in 2021: Integrating GPCR sequence, structure and function. *Nucleic Acids Research*, *49*(D1), D335–D343.

Munk, C., Isberg, V., Mordalski, S., Harpsøe, K., Rataj, K., Hauser, A. S., Kolb, P., Bojarski, A. J., Vriend, G., & Gloriam, D. E. (2016). GPCRdb: the G protein-coupled receptor database – an introduction. In *British Journal of Pharmacology* (pp. 2195–2207). John Wiley and Sons Inc. https://doi.org/10.1111/bph.13509
